# Vermiculite as an Eco-Friendly Catalyst in the Isomerization and Cyclization of Geraniol: Optimization Using the Response Surface Method

**DOI:** 10.3390/molecules30204113

**Published:** 2025-10-16

**Authors:** Anna Fajdek-Bieda, Agnieszka Wróblewska, Mateusz Piz

**Affiliations:** 1 Department of Medical Analytics, Faculty of Health Sciences, Jakub’s from Paradyż Academy in Gorzów Wielkopolski, Chopina 52, 66-400 Gorzów Wielkopolski, Poland; 2 Department of Catalytic and Sorbent Materials Engineering, Faculty of Chemical Technology and Engineering, West Pomeranian University of Technology in Szczecin, Piastów Ave. 42, 71-065 Szczecin, Poland; 3 Department of Inorganic and Analytical Chemistry, Faculty of Chemical Technology and Engineering, West Pomeranian University of Technology in Szczecin, Piastów Ave. 42, 71-065 Szczecin, Poland; mateusz.piz@zut.edu.pl

**Keywords:** geraniol transformations, vermiculite, natural catalysts, sustainable heterogeneous catalysis, green chemistry, XRD, FTIR and SEM, response surface methodology (RSM)

## Abstract

The isomerization of geraniol using natural, acid-modified minerals such as vermiculite presents a promising approach aligned with the principles of green chemistry. Vermiculite, a naturally abundant layered silicate mineral, was subjected to the acid activation and thoroughly characterized using X-ray diffraction (XRD), Fourier-transform infrared spectroscopy (FTIR), and scanning electron microscopy (SEM). These methods allowed the evaluation of crystallinity, structural stability, and surface morphology, which are critical parameters in the heterogeneous catalysis. The catalytic performance of the modified vermiculite was examined in the transformation of geraniol under mild conditions. The study systematically investigated the influence of key process parameters—temperature, reaction time, and catalyst content—on the conversion of geraniol and products selectivities. Optimization using the response surface methodology (RSM), enabled the identification of conditions leading to high conversion of geraniol (up to 85%) and allowing us to obtain favorable selectivities toward linalool, thunbergol, and 6,11-dimethyl-2,6,10-dodecatrien-1-ol. The results indicate that the acid-treated vermiculite exhibits sufficient surface acidity to effectively catalyze isomerization and cyclization reactions, without requiring additional promoters or metal-based systems. Moreover, the use of RSM provided the efficient framework for optimization reaction conditions, reducing experimental workload, and enhancing process efficiency. This study demonstrates the viability of natural, low-cost minerals as environmentally friendly catalysts and supports their integration into sustainable and “green” chemical technologies.

## 1. Introduction

The development of sustainable and green chemical processes is a priority in the modern industrial organic chemistry, where increasing emphasis is placed on the minimalization of the environmental impact, the reduction the application of hazardous reagents and promotion of the use of renewable raw materials [1,2]. Green chemistry principles recommend the replacement of conventional, often toxic catalysts and solvents with environmentally benign alternatives, such as natural minerals and compounds of natural origin. In this context, the development of modern, efficient, cost-effective, and environmentally friendly catalytic systems is becoming increasingly important. This will contribute to achieving sustainable development goals, minimizing energy consumption, and reducing chemical waste [3,4].

A promising avenue of research involves the utilization of the naturally derived catalysts for the transformation of organic compounds, including terpenoids—bioactive molecules extensively applied in the fragrance, pharmaceutical, and cosmetic sectors [5,6]. Among such transformations, the isomerization of geraniol (connected very often with cyclization)—a naturally occurring monoterpenoid alcohol—constitutes the reaction of notable importance in the context of the sustainable chemistry. The conversion of geraniol into more thermodynamically stable and functionally diverse products, including nerol, linalool, β-pinene, and farnesol, enhances the value of this natural compound while enabling the development of greener production pathways for high-demand fragrance and therapeutic compounds [7].

Laboratory-scale investigations play a pivotal role in the advancement of sustainable chemical transformations, offering a controlled setting for the evaluation of novel catalytic materials, optimization of reaction parameters, and assessment of product selectivity and yield. The insights gained from these experiments are critical for the development of scalable and environmentally benign processes, facilitating their translation into industrial applications consistent with the principles of the green and sustainable chemistry [8,9].

**Figure 1:**
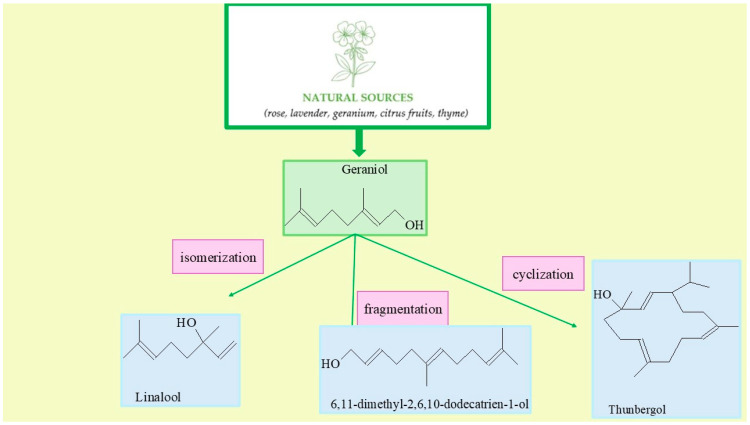
Natural sources of geraniol (rose, lavender, geranium, citrus fruits, thyme) and its main bioconversion pathways. Geraniol can undergo isomerization to linalool, fragmentation to 6,11-dimethyl-2,6,10-dodecatrien-1-ol, or cyclization to thunbergol.

Geraniol (C_10_H_18_O) is the naturally occurring monoterpenoid alcohol, which is present in numerous plants such as rose, lavender, geranium, citrus fruits, and thyme (Figure 1). It is a major component of essential oils extracted from these plants and is widely utilized in various sectors due to its pleasant floral scent and favorable biological properties. In the fragrance industry, geraniol is among the most frequently used ingredients, playing a vital role in the formulation of perfumes, cosmetic products, and air fresheners. Its capacity to enhance and harmonize other scents makes it a key element in both traditional and contemporary fragrance compositions [10,11,12,13,14,15,16,17,18].

In cosmetics, geraniol exhibits moisturizing, anti-inflammatory, antibacterial, and soothing properties. It is employed in a variety of personal care products, including lotions, creams, toners, and hair care formulations. Geraniol anti-inflammatory and antimicrobial effects make it particularly suitable for products targeting sensitive skin or sun exposure damage. Moreover, its aromatherapeutic and stress-relieving qualities contribute to its application in anti-aging and wellness cosmetics [18,19,20].

The pharmaceutical relevance of geraniol is based on its broad biological activity. Studies have shown that it possesses anti-inflammatory, antibacterial, antifungal, and antiviral properties, which allow its use in treating skin conditions such as acne, wounds, burns, and dermatitis. Its therapeutic action also includes improving skin condition by the supporting of regeneration and enhancing skin elasticity, which makes it the popular ingredient in anti-aging formulations [21,22].

Due to its versatility and numerous properties, geraniol plays a key role in many industrial sectors; however, its potential is not limited to these applications. The increasing demand for natural substances in the chemical industry, as well as growing expectations for environmentally friendly production methods, have led to extensive scientific research aimed at discovering new, more efficient ways to process geraniol. One of the most significant modification processes of geraniol is its isomerization, which involves converting this compound into isomers such as nerol, which possess different fragrance, chemical, and biological properties [23,24].

However, the transformation of geraniol can lead to a range of structurally diverse isomers and derivatives whose chemical and sensory properties can be tailored for specific applications. In this study, particular emphasis is placed on optimizing the selective conversion of geraniol into three key products: linalool, 6,11-dimethyl-2,6,10-dodecatrien-1-ol, and thunbergol. These compounds represent distinct structural classes and exhibit varying functional properties, making them valuable in both industrial and research contexts.

Linalool is the unsaturated terpene alcohol with the characteristic floral-lavender scent. It is widely used in cosmetics and pharmaceuticals, valued especially for its calming, antimicrobial, and soothing properties. As a result, it is frequently included in the skincare products and therapeutic formulations [25]. 6,11-Dimethyl-2,6,10-dodecatrien-1-ol is the important intermediate in the synthesis of more complex terpenoids. It serves as a precursor for the production of fragrance compounds and biologically active molecules with the potential applications in perfumery and medicinal chemistry [26]. Thunbergol, the cyclic alcohol, possesses the distinctive aroma and shows promise as the component of natural fragrance blends or plant-based extracts with the potential biological activity. Thunbergol’s unique structure and olfactory profile increase its appeal in perfumery and the development of nature-inspired medicinal and cosmetic formulations [27].

In addition to these target compounds, the transformation of geraniol can also be directed toward the formation of other derivatives such as nerol, β-pinene, and farnesol, which offer further functional benefits. Nerol, for instance, exhibits greater resistance to oxidation and improved thermal stability compared to geraniol, making it suitable for long-lasting fragrance formulations and products exposed to varying environmental conditions [28,29]. On the other hand, β-pinene and farnesol are known for their antimicrobial properties and their ability to enhance fragrance complexity and longevity. These features contribute to their growing relevance in eco-friendly and functional cosmetic and fragrance formulations [30,31,32,33,34].

The isomerization of geraniol not only provides access to compounds with differentiated olfactory and physicochemical characteristics but also offers significant benefits in terms of solubility, UV resistance, and bioactivity—features that are particularly desirable in sun care, medicinal, and therapeutic product lines. From both ecological and economic perspectives, advancing the isomerization process using natural catalysts aligns with sustainable development goals. Natural minerals such as vermiculite offer a promising alternative to synthetic catalysts, supporting the transition toward more environmentally benign and cost-effective industrial practices [12,26].

Among natural catalytic materials, vermiculite—a phyllosilicate mineral with a layered structure—has drawn considerable attention due to its high surface area, chemical stability, and presence of acidic active sites. These characteristics make it the effective and low-cost catalyst for such reactions as the isomerization of geraniol. Its expandable, porous structure enables the incorporation of functional ions and molecules, further enhancing its catalytic performance. Additionally, this mineral composition—vermiculite is rich in aluminum and silicon oxides—facilitates proton transfer and acid–base interactions, which are essential in the mechanism of isomerization of olefinic compounds [35,36,37].

The catalytic function of vermiculite is driven by the presence of active acid centers (e.g., –OH, –Al–OH groups), which mediate protonation–deprotonation processes that lead to the isomers formation. These sites enable the selective conversion of geraniol to nerol, linalool, β-pinene, farnesol, and other valuable compounds under mild conditions. Moreover, vermiculite is reusable, environmentally friendly material, and can be chemically modified to further enhance its catalytic behavior [36].

The optimization of the catalytic systems is crucial to improving selectivity, yield, and raw material efficiency. Response Surface Methodology (RSM) is an established tool for reaction optimization that allows simultaneous evaluation of multiple process parameters, such as temperature, reaction time, and catalyst amount. Through modeling and statistical analysis, RSM facilitates the identification of optimal operating conditions that minimize resource consumption and maximize output—key criteria for sustainable industrial applications [38,39,40].

The purpose of this work is to evaluate the potential of vermiculite as the catalyst in the process of the isomerization of geraniol, and to optimize the conditions of the process to increase its efficiency and products selectivities. Specifically, the goal is to study the catalytic properties of vermiculite, including its structure, acid activity and role in isomerization reactions, and to optimize key reaction parameters, such as temperature, reaction time and content of the catalyst, using modern optimization methods such as the response surface method (RSM). The work aims to develop optimal reaction conditions to maximize the yields of the main geraniol derivatives studied here—linalool (LO), 6,11-dimethyl-2,6,10-dodecatrien-1-ol (DCM), and thunbergol (TH)—while minimizing raw material consumption. In addition, the goal is also to explore the potential of vermiculite as the environmentally friendly and low-cost catalyst in the chemical industry, which could provide the alternative to traditional synthetic catalysts used in similar processes.

## 2. Results

### 2.1. Characteristics of Vermiculite with Instrumental Methods

**Figure 2:**
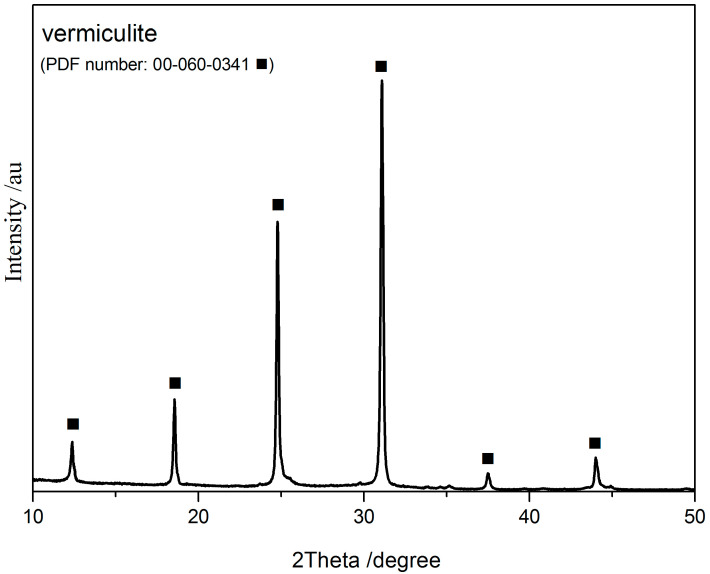
XRD pattern of vermiculite sample.

In the first stage of the study, the sample of vermiculite was analyzed using X-ray diffraction. The obtained XRD pattern of vermiculite sample is presented in Figure 2.

The XRD pattern of vermiculite (Figure 2) exhibits distinct diffraction peaks at 12.3°, 18.3°, 25.1°, 32.2°, 37.6° and 44.8° 2θ, corresponding to the reference PDF number 00-060-0341. The strongest peak, observed at 2θ ≈ 31.0°, can be assigned to the (004) plane, characteristic of well-ordered 2:1 layered silicate minerals. No diffraction peak is observed around 39°, confirming the absence of additional phases such as montmorillonite, illite, or kaolinite. The high intensity and narrow full width at half maximum (FWHM) of the peaks indicate good crystallinity, a single-phase nature, and preserved structural integrity of the vermiculite. The observed reflections correspond to basal layer spacings (d-spacings), which are relevant for potential applications in sorption, catalysis, or surface modification. Overall, the pattern confirms that the vermiculite structure is stable, with no intercalation, interlayer expansion, or amorphization [37,38].

**Figure 3:**
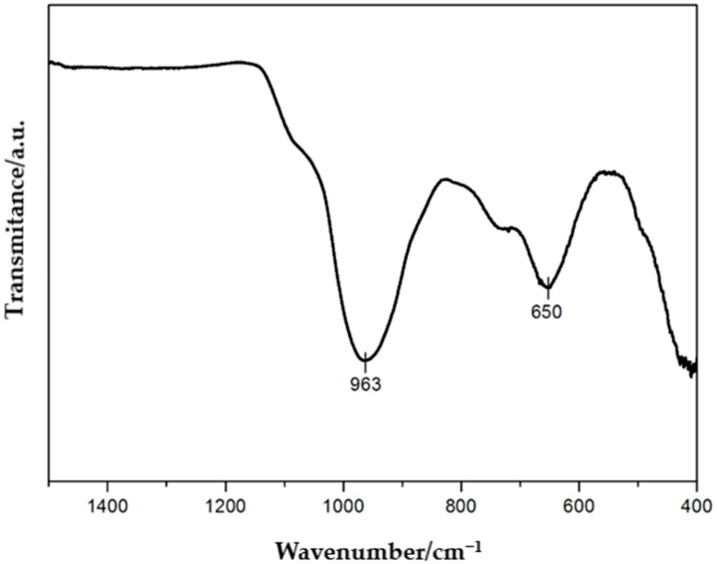
FTIR spectrum of vermiculite.

The FTIR spectrum (Figure 3) recorded for the vermiculite sample in the range of 400–1500 cm^−1^ reveals characteristic absorption bands that clearly confirm the ordered layered structure of this mineral. The most intense and diagnostic band is the band observed at 963 cm^−1^ which corresponds to the asymmetric stretching vibrations of Si–O bonds within the tetrahedral network of the aluminosilicate. This band is typical for vermiculite and other layered clay minerals and is directly related to the fundamental tetrahedral structure composed of SiO_4_^4−^ units. Another significant band in this spectrum appears around 650 cm^−1^ and can be attributed to bending (deformation) vibrations of the Si–O–Mg or Si–O–Al bonds present in the octahedral layers. The presence of this signal suggests a well-preserved octahedral network as well as the presence of Mg^2+^ and Al^3+^ cations in the layered structure, consistent with the typical chemical composition of vermiculite. The analyzed spectrum is characterized by the absence of intense signals in the range of 800–1200 cm^−1^, which excludes the presence of organic contaminants as well as amorphous silicates or carbonates. This indicates the high mineralogical purity of the sample and the lack of secondary phases that could indicate degradation or modification processes. Furthermore, the absence of bands in the hydroxyl group region (i.e., 3400–3700 cm^−1^) within the analyzed range may be due to the limited spectral range or the low content of interlayer water.

In summary, the presented FTIR spectrum confirms the presence of the pure vermiculite phase with a well-preserved tetrahedral–octahedral structure. The spectroscopic analysis shows no evidence of impurities or degradation products, indicating the high quality of the studied sample. The obtained data can serve as the reference point for further studies on the chemical modifications, intercalation or surface functionalization of this mineral [37,38].

**Figure 4:**
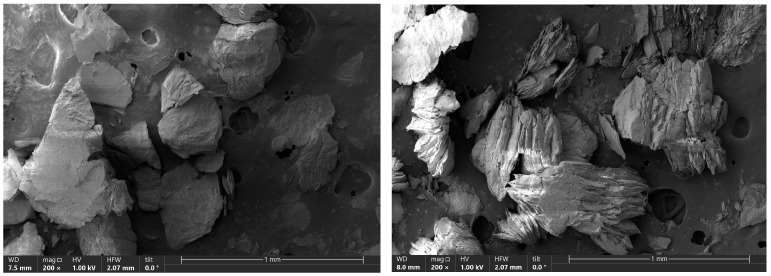
The SEM images of the vermiculite.

The presented scanning electron microscope (SEM) images (Figure 4), taken at 200× magnification, reveal the morphology of natural vermiculite, a mineral characterized by the distinctive layered structure. The surface displays irregular, flaky fragments of varying sizes, loosely arranged against a porous substrate. This structure is typical for layered minerals, where the arrangement of tetrahedral and octahedral layers (SiO_4_, Mg^2+^, Fe^3+^, Al^3+^) creates interlayer spaces that promote swelling and adsorption of reagents. The visible cracks and pores in the material indicate the high specific surface area of the vermiculite, which is crucial for its catalytic properties. The layered structure, along with the presence of surface acid sites, enables the effective protonation of substrates such as geraniol, leading to the formation of stable allylic carbocations—key intermediates in the isomerization and cyclization reactions [37,38].

Both the morphology and porosity observed in the images confirm the ability of vermiculite to form localized reaction microenvironments (microreactors) that restrict the rotational freedom of intermediate carbocation forms and stabilize transitional reaction complexes. Such conditions favor the selective synthesis of products like linalool, thunbergol, and 6,11-dimethyl-2,6,10-dodecatrien-1-ol. In our earlier article [20], we presented the results of a comparative determination of total acidity for vermiculite and two well-known catalysts, TS-1 and ZSM-5, which have long been used in the organic industry. This determination indicates that vermiculite is a material characterized by a slightly lower total acidity than the industrially known catalysts TS-1 and ZSM-5—the acid-site concentration expressed in [mmol/g] for TS-1 is 1490, for ZSM-5 1475, and for vermiculite 1049. This does not mean, however, that vermiculite will be an inferior catalyst to TS-1 and ZSM-5 catalysts. The ability of reactants to diffuse into the pores and, consequently, the availability of the active centers in the pores to the reacting molecules will be of great importance, as will the residence time of the products in the pores, as prolonged residence of reactant and product molecules in the pores can lead to the formation of by-products.

### 2.2. Preliminary Studies on the Isomerization of Geraniol on Vermiculite

The preliminary studies on the isomerization of geraniol evaluated the effects of temperature, catalyst content and reaction time on the main products selectivities and the conversion of GA. The main reaction products, which were detected in the post-reaction mixtures, were linalool (LO), 6,11-dimethyl-2,6,10-dodecatrien-1-ol (DCM) and thunbergol (TH). Table 1 shows the results of experiments carried out at different temperatures, and at the constant values of reaction time (3 h) and amount of the catalyst (5 wt%). The aim of this stage of the study was to determine the best conditions for the obtaining maximum values of the selectivities of the main products while achieving high GA conversion.

**Figure 5:**
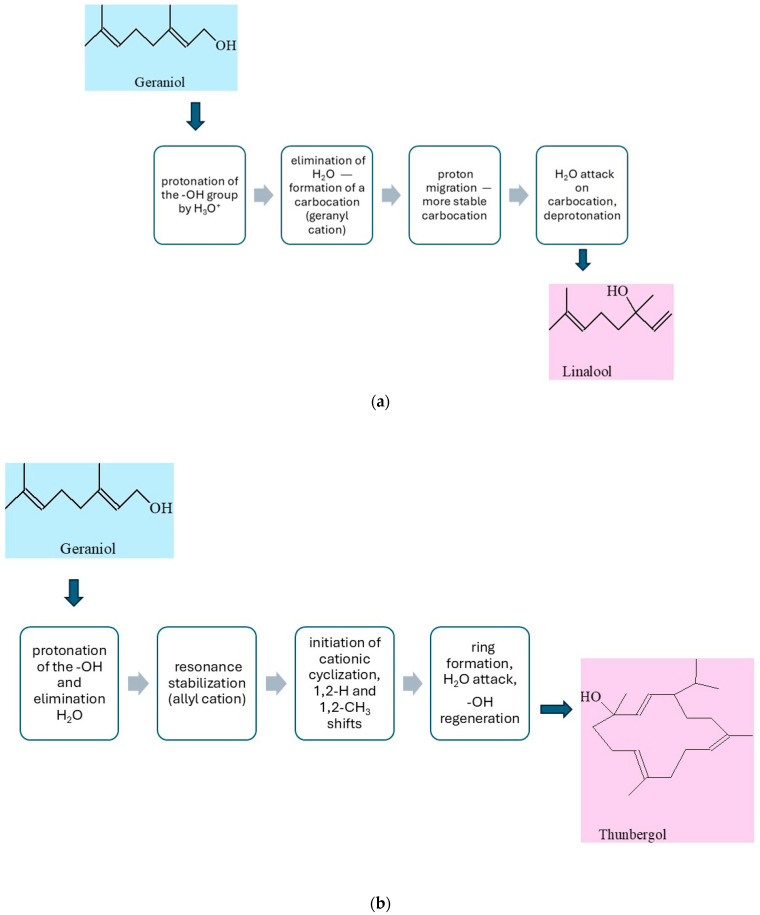

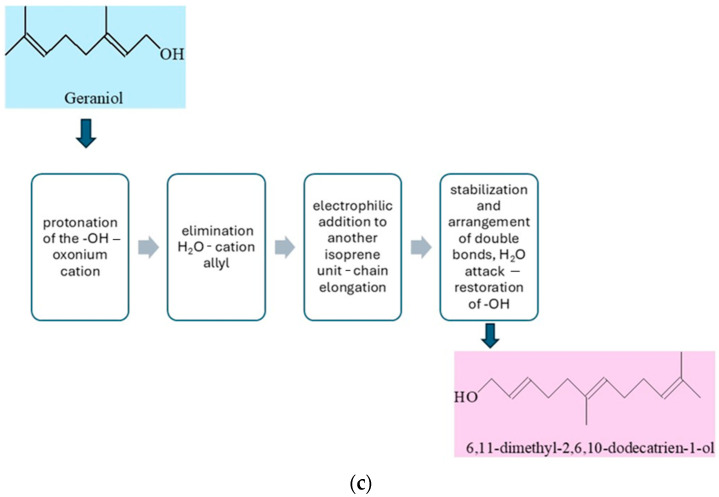
The reaction pathway of geraniol transformation on vermiculite (**a**) isomerization of geraniol to linalool, (**b**) cyclization of geraniol to thunbergol, (**c**) condensation of geraniol to 6,11-dimethyl-2,6,10-dodecatrien-1-ol.

The possible reaction pathway of geraniol transformation on vermiculite is presented in Figure 5a–c.

The simplified mechanism of isomerization of geraniol to linalool (LO) is presented in Figure 5a. Under acidic conditions, geraniol, an unsaturated monoterpene alcohol, is converted to its structural isomer, linalool, in a reaction involving a cationic mechanism. This process begins with the protonation of the hydroxyl group of geraniol by a hydronium ion (H_3_O^+^), resulting in the formation of the –OH_2_^+^ group, which is a good leaving group. This is followed by the elimination of a water molecule, leading to the formation of a carbocation—the so-called geranyl cation. The resulting carbocation is stabilized by resonance within the system of conjugated double bonds, which allows for its reorganization. Next, isomerization of the cation occurs, leading to rearrangement of the π bond system and proton migration, resulting in the formation of a more stable tertiary carbocation in the position adjacent to the end of the side chain. At this stage, a nucleophilic attack of a water molecule on the electrophilic center (carbene cation) occurs, leading to the formation of an intermediate protonated alcohol. Finally, through deprotonation, the final product is obtained—linalool, which is an unsaturated aliphatic alcohol with a different position of the hydroxyl group compared to the starting geraniol [41].

In the presence of the solid vermiculite catalyst, the transformation of geraniol into thunbergol (Figure 5b) proceeds through several stages via an acid-catalyzed cationic cyclization typical of terpenoid compounds. Vermiculite, a layered aluminosilicate, contains both Brønsted acid sites (protonating) and Lewis acid sites (coordinating), which enable activation of the hydroxyl group of geraniol. In the first stage, geraniol condenses with isoprene units at these acidic sites, forming trans,trans-farnesol and then trans,trans,trans-geranylgeraniol. The adsorbed geranylgeraniol is protonated at the oxygen atom, forming an oxonium species, followed by elimination of water and generation of a resonance-stabilized allylic carbocation at the C-1 position. This carbocation initiates a cascade of intramolecular electrophilic additions along the side chain. After the formation of the first ring, the carbocation migrates via 1,2-hydride or 1,2-methyl shifts, allowing further cyclizations and construction of the thunbergol ring system. The layered structure of vermiculite stabilizes intermediates and enforces a molecular conformation favorable for selective cyclization. In the final step, the terminal carbocation is quenched by nucleophilic attack of a water molecule from the catalyst surface, restoring the hydroxyl group, and the final thunbergol product detaches into the liquid phase. This non-enzymatic process under heterogeneous catalysis ensures high selectivity and efficiency [42].

The conversion of geraniol to 6,11-dimethyl-2,6,10-dodecatrien-1-ol in the presence of a solid acid catalyst such as vermiculite proceeds via a cationic acid-catalyzed condensation mechanism characteristic of reactions forming longer isoprenoid chains (Figure 5c). The geraniol molecule adsorbs onto the catalyst surface in the vicinity of an acid site. Subsequently, the hydroxyl group of geraniol undergoes protonation at the oxygen atom, leading to the formation of an unstable oxonium cation. In the next step, a water molecule is eliminated, resulting in the formation of a resonance-stabilized allylic carbocation at the C-1 position. This cation acts as an electrophile and attacks the double bond of another isoprenoid molecule or a chain fragment within the same molecule, causing chain elongation toward the formation of the 6,11-dimethyl-2,6,10-dodecatriene skeleton. The newly formed carbocation may undergo 1,2-hydride or 1,2-methyl shifts, which stabilize the structure and lead to the appropriate arrangement of double bonds. The process concludes with quenching of the carbocation by nucleophilic addition of a water molecule originating from the catalyst surface, thereby regenerating the hydroxyl group at the C-1 position. The layered structure of vermiculite stabilizes reaction intermediates and enforces a substrate conformation favorable for the selective course of condensation. The reaction proceeds under conditions of heterogeneous acid catalysis, which ensures high selectivity and efficiency, minimizes the formation of by-products, and enables the effective synthesis of 6,11-dimethyl-2,6,10-dodecatrien-1-ol from geraniol [42].

Temperature plays a key role in the kinetics and mechanism of the organic reaction. Increasing the temperature affects not only on the rate of reaction, but also on the course of reaction pathways, including the stability and isomerization of carbocation intermediates. Geraniol, as the terpene alcohol with the unsaturated system, can undergo a variety of transformations, many of which are initiated by the protonation or electrophilic activation.

At 50 °C, GA conversion was 84.6 mol% and the selectivity of such products as LO, DCM and TH was relatively low. This can be attributed to insufficient activation energy for more complex structural reorganizations of the GA molecule, such as cyclization or conformational transformations that lead to more extended terpenes such as DCM and TH.

At 60 °C, a slight increase in selectivity is already observed for linalool—a monoterpene with the acyclic structure, with a single hydroxyl group and a conjugated double-bond system. Linalool is mainly formed by the isomerization of geraniol, and higher temperature may promote the transition state needed to change the configuration of double bonds (e.g., from trans- to cis-) and migration of the hydroxyl group.

At 70 °C, there is a significant increase in LO selectivity to 15.81 mol%, which correlates with the increase in GA conversion to 91.98 mol%. This temperature promotes the formation of thermodynamically more stable products, such as LO, which has a relatively low free energy of the final form. Isomerization and transformation reactions of allylic functional groups become more efficient, which may suggest the involvement of the SN1 mechanism or reactions involving carbocation intermediates.

At 80 °C, GA conversion reaches a maximum value (99.6 mol%), and LO selectivity stabilizes at 15.73 mol%. Importantly, under all tested conditions, the selectivity toward thunbergol (TH) remained the highest among all products, highlighting that the reaction pathway leading to TH is strongly favored at moderate to high temperatures, likely due to the stability of the carbocation intermediates involved in the cyclization steps. The low selectivities for DCM indicate that its formation requires more complex, multi-step transformations involving tertiary carbocations or cyclic intermediates, which are less favored thermodynamically under these conditions.

The temperature of the reaction performing affects not only the rate of GA conversion, but also the nature of the resulting products in terms of their chemical structure and termodynamic stability. The increase in the temperature favors the formation of simple acyclic product (linalool), while more complex cyclic products (such as TH) may require more demanding conditions or the presence of specific catalysts directing the reaction pathway. Process optimization should therefore take into account not only selectivity and conversion, but also mechanistic analysis of the reaction, taking into account the stability of carbocation intermediates, enthalpies of cyclization and steric effects related to the structure of the final products.

In the next series of experiments during our preliminary studies, the effect of catalyst content (vermiculite) on the selectivities of the appropriate products and geraniol conversion was analyzed. The experiments were carried out at the constant reaction temperature (70 °C) and for the constant reaction time (3 h), with the changing in the catalyst content in the range of 1 wt% to 10 wt% (Table 2).

The obtained data indicate that the increase in the catalyst content leads to significant changes in both the selectivity of individual products and the conversion of GA.

At the lowest vermiculite content (1 wt%), the GA conversion reached 89.12 mol%, with dominant selectivity toward thunbergol (TH, 26.48 mol%) and moderate selectivity for linalool (LO, 14.94 mol%). The low selectivity for 6,11-dimethyl-2,6,10-dodecatrien-1-ol (DCM, 0.32 mol%) suggests that at such low catalytic activity, the reaction predominantly yields simpler products, whose formation requires less involvement in cyclization and condensation processes.

The increase in the catalyst content to 2.5 wt% results in the slight increase in GA conversion (89.76 mol%) and the moderate increase in the selectivity of TH (28.31 mol%) and DCM (0.43 mol%), while the selectivity of LO remains at the similar level (14.42 mol%). This suggests that at this catalyst content, additional reaction pathways leading to structurally more complex products—such as the sesquiterpene thunbergol—begin to be activated.

With the application of 5 wt% of the catalyst, a further increase in the selectivity of main products is observed—DCM selectivity increases to 2.82 mol% and TH to 37.70 mol%, indicating enhanced catalytic surface activity and the promotion of condensation, addition and cyclization reactions. Linalool remains the important reaction product (the selectivity of this compound amounts to 15.81 mol%), though its share no longer increases significantly, suggesting that under these conditions, competing reaction pathways start to dominate.

At higher catalyst contents (7.5 wt% and 10 wt%), there is a significant shift in the reaction equilibrium towards the formation of high-molecular-weight and more structurally complex products. The selectivity of TH increases accordingly with 60.86 mol% and 65.18 mol%, while LO remains at a high level (22.71 mol% and 24.77 mol%, respectively). The selectivity of DCM stays at the moderate level (2.34–2.06 mol%), suggesting that its formation is not the main reaction pathway, even at higher catalyst content.

Increasing the catalyst content in the reaction mixture leads to higher GA conversion and promotes the formation of more complex products, mainly cyclic ones (such as TH), through the activation of cyclization and condensation reactions. The high selectivity for TH along with the high GA conversion at the content of the catalyst ≥ 7.5 wt%, indicates the high potential of vermiculite as the acidic catalyst for sesquiterpene formation. At the same time, a limited increase in LO selectivity is observed, suggesting that its formation mainly occurs in the early stages of the reaction performing and it is not the dominant product under high catalyst content. Therefore, the optimal catalyst content should be selected depending on which final product—linalool or thunbergol—is preferred.

In the conducted preliminary studies, the influence of reaction time on the course of geraniol (GA) transformation in the presence of vermiculite as the catalyst was also analyzed. The reactions were carried out at the constant temperature of 70 °C, with the catalyst content of 10 wt% in the reaction mixture, while changing the reaction time from 15 min to 24 h. Changes in products selectivities and GA conversion were observed in an effort to determine the most beneficial reaction time that would ensure high selectivity of the desired products—linalool (LO), 6,11-dimethyl-2,6,10-dodecatrien-1-ol (DCM), and thunbergol (TH)—while simultaneously achieving maximum substrate consumption (Table 3).

After just 15 min, a very high GA conversion was observed, reaching 99.52 mol%. At this time, the selectivity for LO was low and amounted to 5.7 mol%, whereas TH was formed in significantly greater amounts—its selectivity reached 14.62 mol%. The share of DCM was trace (0.04 mol%). This indicated that the cyclization of geraniol to TH occurred very rapidly, while the isomerization to LO and condensation to DCM proceeded more slowly.

With the prolongation of the reaction time to 0.5 h, a clear increase in LO and TH selectivities was observed—LO selectivity increased to 13.28 mol%, and TH to 53.29 mol%. GA conversion remained at a very high level (99.54 mol%), and the content of DCM increased only slightly (0.48 mol%). After one hour of reaction performance, LO selectivity reached 19.73 mol%, and TH selectivity amounted to 54.10 mol%. The increase in DCM selectivity reached 0.73 mol%. This indicated further development of isomerization and condensation pathways, while the catalyst maintained high activity.

After two hours the increase in the selectivity of LO was also observed, and this function reached value 20.74 mol%, and TH selectivity was 57.89 mol%. DCM was also formed in greater amounts (DCM selectivity was 1.66 mol%). After three hours, LO selectivity continued to rise and reached its maximum value of 24.77 mol%, while TH dominated in the post-reaction mixture and its selectivity amounted to 65.96 mol%. DCM selectivity achieved value of 2.06 mol%. At this reaction time, GA conversion was practically complete, reaching 99.81 mol%. It was therefore observed that the prolongation the reaction time to 3 h favored both the obtaining maximum value of GA conversion and the increase in selectivity of the desired reaction products, especially linalool and thunbergol.

After exceeding a reaction time of 3 h (i.e., at 4 h and beyond), significant alterations in the composition of the post-reaction mixture were observed. Gas chromatography (GC) analysis revealed the absence of previously detected compounds such as LO, DCM, and TH, indicating their complete transformation. Despite the lack of detectable signals for these compounds, visual examination of the reaction mixture suggested the onset of secondary processes, primarily degradation and oligomerization. These reactions appeared to intensify under the influence of the acidic catalyst and the sustained high temperature. The uncontrolled transformation of molecules containing unsaturated bonds led to the formation of high-molecular-weight products, including heavy, often insoluble oligomers and resinous materials.

In summary, as the reaction time increased, a systematic rise in the selectivity of the desired products—LO and TH—was observed, reaching maximum values after approximately 3 h. Beyond this point, these products began to undergo secondary transformations, leading to the formation of undesirable by-products. The most beneficial reaction time was therefore determined to be 3 h. Under these conditions, high selectivity toward LO and TH was achieved, accompanied by nearly complete conversion of GA, without any noticeable signs of degradation or polymerization of the target products.

The absence of LO, DCM, and TH after extending the reaction time to 4 h and further up to 24 h results from secondary reactions catalyzed by the acidic sites affecting these products. Under prolonged contact with the acidic sites of vermiculite at 70 °C, LO, DCM, and TH undergo further reactions, such as intermolecular electrophilic additions, oligomerization, and condensation, leading to the formation of high-molecular-weight polymeric species. These reactions compete with the primary transformation of geraniol, effectively consuming the initially formed monoterpenoids. This observation indicates that extended reaction times favor polymerization at the expense of accumulation of individual monomeric products.

### 2.3. Optimization the Process of Isomerization of Geraniol on Vermiculite

#### 2.3.1. Influence of Process Parameters on GA Conversion

To thoroughly investigate the influence of key process parameters—temperature, catalyst content and reaction time on the geraniol conversion and the selectivity of the appropriate products, a statistical analysis of the experimental data was performed using multiple regression and analysis of variance (ANOVA), with a significance level set at 95% (α = 0.05). The regression model was used to develop an equation describing the behavior of the system as a function of the three independent variables, as well as to evaluate the interactions between them (Table 4).

Based on the data presented in Table 5, the model demonstrates a very good fit to the experimental data: the coefficient of determination R^2^ = 96.92%, adjusted R^2^ = 95.28%, and predicted R^2^ = 92.17%. These values clearly indicate that the model accurately describes the observed reaction system and can be effectively used for its prediction and optimization. The high value of adjusted R^2^ confirms that a significant portion of the variability in the response (GA conversion) is attributable to the variability of the applied factors (parameters).

The analysis of variance shows that all three linear factors (parameters)—reaction temperature (F = 465.36; *p* = 0.000), catalyst content (F = 41.90; *p* = 0.000), and reaction time (F = 22.66; *p* = 0.000)—have a statistically significant effect on GA conversion. The remaining components of the model, i.e., the quadratic terms and two-factor interactions, did not reach statistical significance (*p* > 0.05), indicating that within the tested range their influence on the response was negligible. This result suggests that the system behaves in an approximately linear manner, which simplifies further analysis and prediction. Additionally, the variance inflation factors (VIF = 1.00 for all variables) confirm the absence of multicollinearity among the independent variables, which positively affects the reliability of the regression model estimation (Table 5).

Regression Equation in Uncoded Units:

(1) $$C_{GA}=80.35+0.0582\;T+0.228\;C+0.0651\;\tau -0.000027\;T^{2}-0.0012\;C^{2}-0.00124\;{\;\tau }^{2}\;-0.000820\;T\cdot C\;-0.000561\;T\cdot \tau \;-0.00392\;C\cdot \tau \;$$

where

*C_GA_* is conversion of geraniol [wt%],

*T* is temperature [°C],

*C* is catalyst content [wt%],

*τ* is reaction time [h].

From the above equation, it follows that temperature has the greatest impact on geraniol conversion, as indicated by the highest linear coefficient. This is related to both the kinetics of the oxidation process and the activity of the catalytic center, which in this case is strongly temperature dependent. Reaction time shows a moderate effect, suggesting that the transformation proceeds relatively quickly and that conversion reaches a plateau within a short time frame.

**Figure 6:**
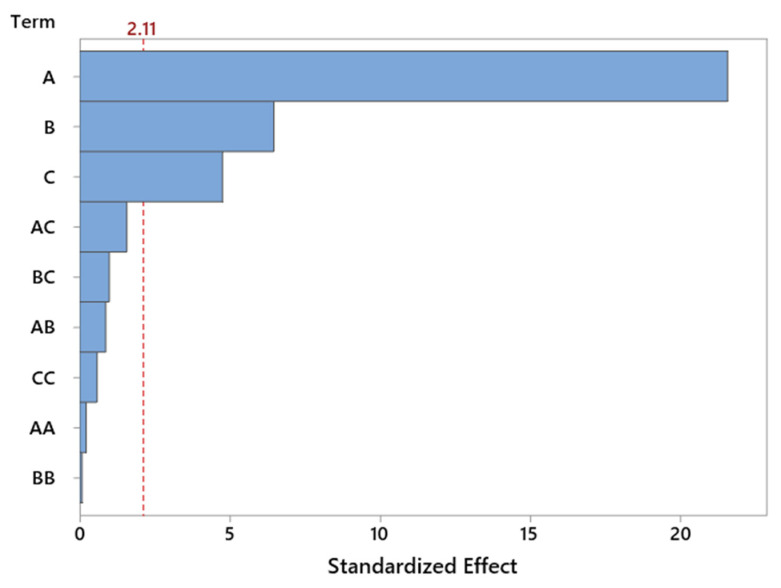
Pareto chart of the standardized effects of geraniol conversion plot for α = 0.05 (A—Reaction temperature, B—Catalyst content (wt%), C—Reaction time (hours), AB—Interaction between reaction temperature and catalyst content, AC—Interaction between reaction temperature and reaction time, BC—Interaction between catalyst content and reaction time, AA—Quadratic effect of reaction temperature (nonlinear effect of temperature), BB—Quadratic effect of catalyst content (nonlinear effect of catalyst content), CC—Quadratic effect of reaction time (nonlinear effect of reaction time)).

The Pareto chart (Figure 6) for standardized effects confirms the dominant influence of reaction temperature, which significantly exceeds the contribution of other variables in terms of statistical significance. The importance of catalyst content is also evident, whereas the interaction effects and nonlinear terms do not reach the threshold of significance. This observation supports the earlier conclusion that the system behaves in a nearly linear manner within the tested range of variables, which is favorable from the standpoint of model predictability and process optimization.

**Figure 7:**
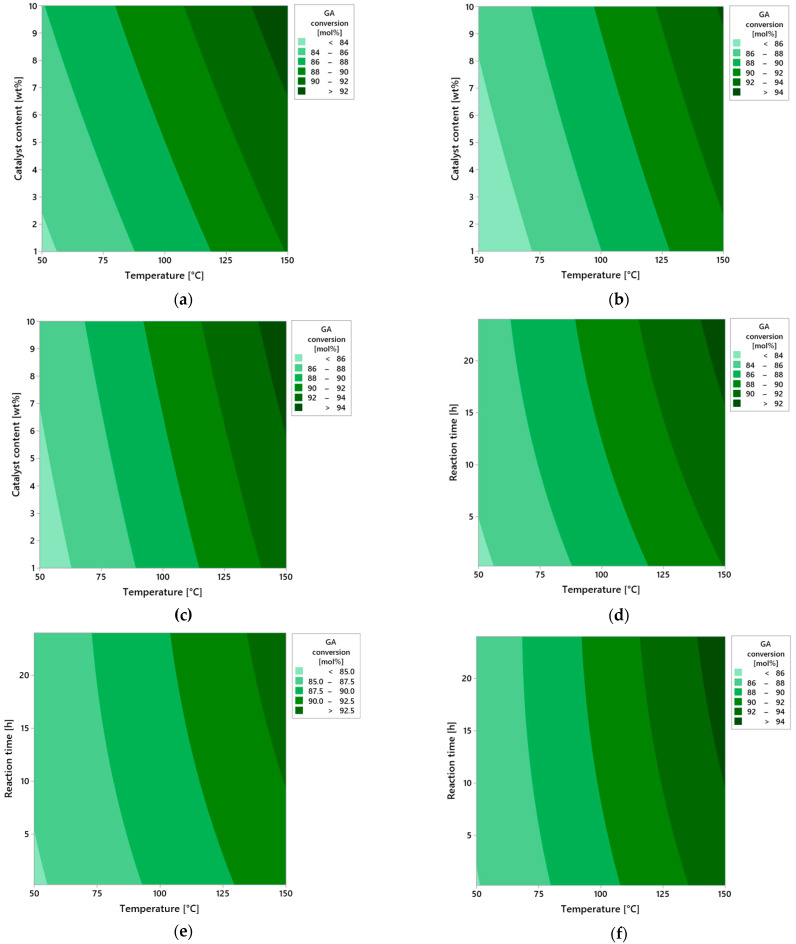

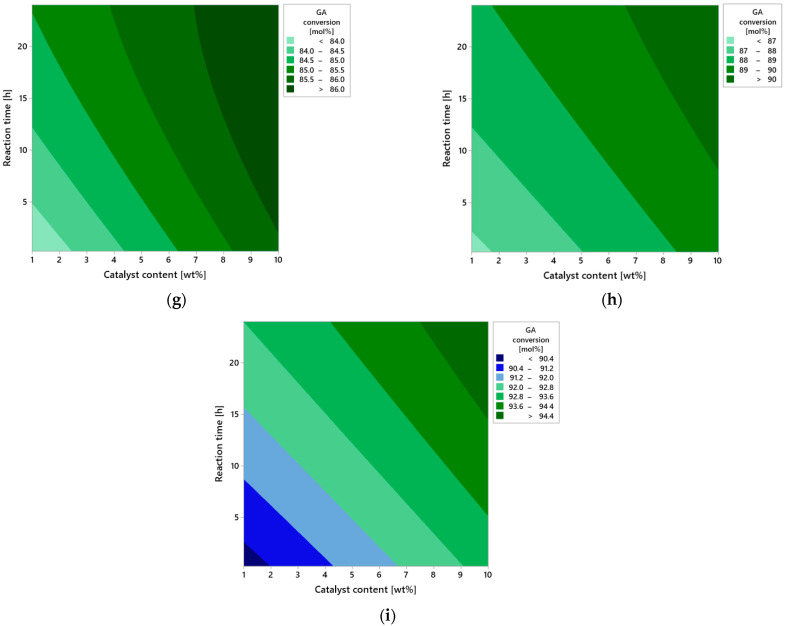
Contour plot of geraniol conversion at: time (**a**) 0.25 h, (**b**) 12 h, (**c**) 24 h, catalyst content (**d**) 1 wt%, (**e**) 5 wt%, (**f**) 10 wt%, temperature (**g**) 50 °C, (**h**) 100 °C, (**i**) 150 °C.

Figure 7 presents a series of contour plots illustrating the influence of three key process parameters—reaction time, catalyst content, and temperature—on the conversion of geraniol (GA) in the oxidation reaction carried out in the presence of vermiculite as the heterogeneous catalyst.

The first group of plots (a–c) analyzes the effect of reaction time: 0.25 h (a), 12 h (b), and 24 h (c). At a very short reaction time (0.25 h), regardless of other process variables, the conversion of geraniol is clearly low, indicating limited contact of the substrate with the active surface of the catalyst. The short residence time does not allow for effective adsorption and activation of geraniol molecules. As the reaction time increases to 12 h, a significant rise in conversion is observed, particularly under moderate temperature conditions (around 100 °C) and medium catalyst loading. The highest conversion, approaching 100 mol%, is achieved at the maximum reaction time of 24 h, under conditions of elevated temperature and increased catalyst content. The extended reaction time enables more complete utilization of the catalytic surface of vermiculite and allows the reaction to proceed to completion with minimal formation of side products.

The second series of plots (d–f) illustrates the effect of catalyst content: 1 wt% (d), 5 wt% (e), and 10 wt% (f). At the lowest catalyst amount (1 wt%), the system shows limited activity—conversion does not exceed 50 mol%, even at higher temperatures and longer reaction times. This results from the insufficient number of active catalytic sites available on the vermiculite surface. Increasing the catalyst content to 5 wt% leads to a noticeable improvement in reaction performance—conversion significantly increases, especially at moderate temperatures and extended reaction durations. A maximum catalyst amount of 10 wt% results in nearly complete conversion of geraniol, confirming the synergistic effect between the amount of active catalytic material and the overall intensity of the process.

The final group of plots (g–i) shows the effect of temperature: 50 °C (g), 100 °C (h), and 150 °C (i). At the lowest temperature studied (50 °C), the reaction proceeds only to a very limited extent—the conversion remains low, likely due to insufficient activation energy for the oxidation process. At 100 °C, a significant increase in conversion is observed—these conditions may be considered optimal from the perspective of balancing selectivity and reaction rate. At 150 °C, maximum conversion is achieved, suggesting that the catalytic system with vermiculite tolerates elevated temperatures well, maintaining both activity and structural stability.

In summary, all three examined parameters—reaction time, catalyst content, and temperature—have a positive influence on geraniol conversion, with temperature appearing to be the most significant. The high catalytic efficiency of vermiculite, despite its simple layered structure, confirms the potential of this natural mineral as the effective catalyst in isomerization reactions of unsaturated alcohols such as geraniol.

#### 2.3.2. Influence of Process Parameters on LO Selectivity

In this study, a detailed analysis was conducted to evaluate the influence of key process parameters—temperature, catalyst content, and reaction time—on the selectivity of LO (LO selectivity), using regression analysis and analysis of variance (ANOVA) at a 95% confidence level (α = 0.05). The resulting statistical model demonstrates a very high degree of fit, as confirmed by the values of the determination coefficients: R^2^ = 99.56%, adjusted R^2^ = 99.33%, and predicted R^2^ = 98.90%. These high indicators clearly demonstrate that the model accurately reflects the relationships between the independent variables and the selectivity of the process (Table 6).

The ANOVA analysis confirmed that all linear factors—temperature, catalyst content, and reaction time—have the statistically significant effect on LO selectivity (*p* < 0.0001). Among these, temperature proved to be the most influential factor, with the F-statistic value as high as 3449.54, clearly indicating its dominant role in determining the efficiency of the reaction. Catalyst content also exhibited a strong influence (F = 219.97), while reaction time, though to a lesser extent, was still statistically significant (F = 126.16). The quadratic terms showed moderate significance: the quadratic effect of temperature had the greatest impact (F = 52.09), whereas the quadratic term for reaction time was statistically insignificant (*p* = 0.987), suggesting that extending the reaction time beyond a certain threshold does not further enhance selectivity. Most of the two-factor interactions were not statistically significant, with the exception of the temperature–reaction time interaction (*p* = 0.004), which may indicate a potential synergistic effect between these two parameters (Table 7).

Regression Equation in Uncoded Units:

(2) $$S_{LO}=-14.03+0.3928\;T+1.016\;C+0.0197\;\tau -0.000956\;T^{2}-0.0519\;C^{2}+0.00004\;{\;\tau }^{2}\;-0.00124\;T\cdot C\;-0.001333\;T\cdot \tau \;-0.00490\;C\cdot \tau \;$$

where

*S_LO_* is LO selectivity [mol %],

*T* is temperature [°C],

*C* is catalyst content [wt%],

*τ* is reaction time [h].

The coefficients of the equation confirm the dominant role of temperature and catalyst content as the main factors shaping the selectivity of LO. The very low coefficients associated with the quadratic and interaction terms additionally indicate a moderate degree of nonlinearity in the system.

**Figure 8:**
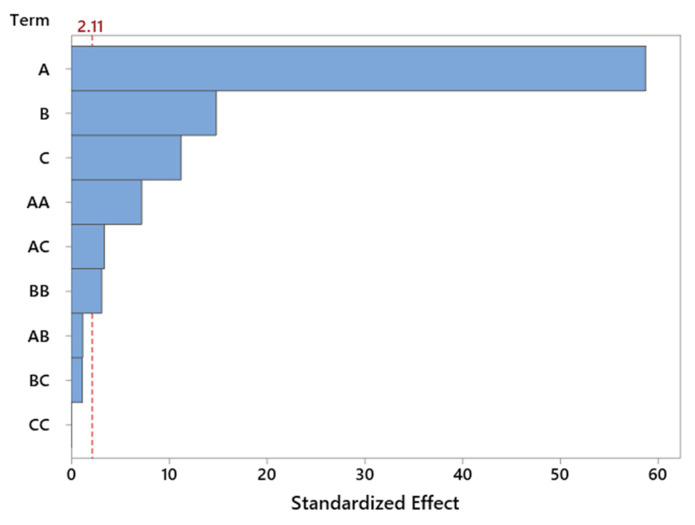
Pareto chart of the standardized effects of LO selectivity plot for α = 0.05 (A—Reaction temperature, B—Catalyst content (wt%), C—Reaction time (hours), AB—Interaction between reaction temperature and catalyst content, AC—Interaction between reaction temperature and reaction time, BC—Interaction between catalyst content and reaction time, AA—Quadratic effect of reaction temperature (nonlinear effect of temperature), BB—Quadratic effect of catalyst content (nonlinear effect of catalyst content), CC—Quadratic effect of reaction time (nonlinear effect of reaction time)).

Graphical analysis in the form of the Pareto chart (Figure 8) clearly confirms the statistical results obtained from the ANOVA analysis. Temperature exhibits the greatest influence on the selectivity of the transformation to LO compound—the bar corresponding to this factor significantly exceeds the others in length, highlighting its dominant role in governing the course of the reaction. This strong significance of temperature may be related to the energetic activation of the reaction system, the increased rate of the main reaction, and the more efficient activation of both reagents and catalytic centers. Higher temperatures also help suppress side reactions, which in turn improves overall selectivity. The second most significant factor is catalyst content, likely due to the greater availability of active sites at higher loadings. On the other hand, the interactions between parameters, although statistically detectable, have a relatively minor influence and do not outweigh the effects of the individual factors (Figure 8).

**Figure 9:**
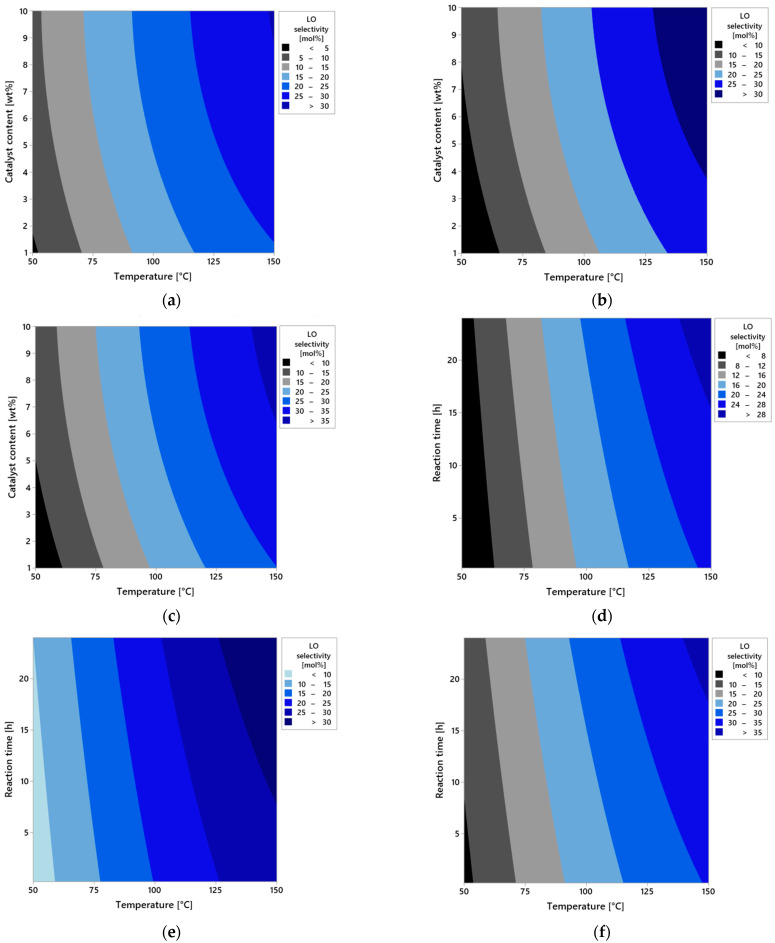

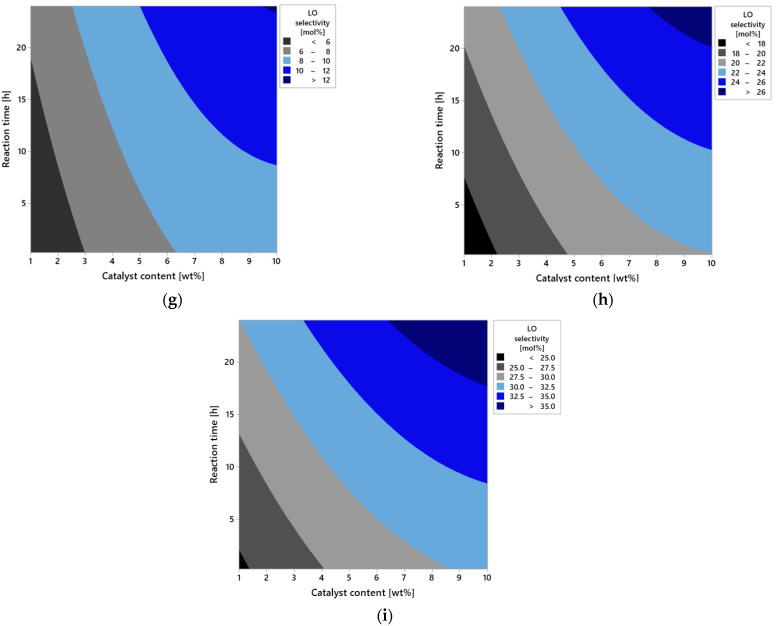
Contour plot of LO selectivity at: time (**a**) 0.25 h, (**b**) time 12 h, (**c**) 24 h, catalyst content (**d**) 1 wt%, (**e**) 5 wt%, (**f**) 10 wt%, temperature (**g**) 50 °C, (**h**) 100 °C, (**i**) 150 °C.

Figure 9a–i present a series of contour plots that visualize the spatial relationships between the process parameters and LO selectivity. In the first set of plots (a–c), the effect of reaction time is illustrated. For a very short reaction time (0.25 h, Figure 9a), the selectivity of the transformation to LO remains low regardless of other conditions. This is due to limited contact time between the substrate and the catalyst, which prevents full activation of the reaction. Increasing the reaction time to 12 h (Figure 9b) results in a significant increase in selectivity of LO, especially at higher temperatures and moderate catalyst content. This suggests the development of the full reaction mechanism over time, where side reactions are more effectively suppressed and the dominant pathway becomes the one leading to LO. At the maximum reaction time (24 h, Figure 9c), near-maximal selectivity of LO is observed under high temperature and high catalyst content conditions. This may indicate that the process reaches a quasi-equilibrium state in which the substrate is predominantly converted via a single dominant reaction mechanism.

In the second series of plots (Figure 9d–f), the effect of catalyst content is presented. At the lowest level (1 wt%, Figure 9d), selectivity remains low, suggesting that the number of active catalytic sites is insufficient for effective conversion of the substrate to the desired product. At a moderate concentration (5 wt%, Figure 9e), an improvement in the selectivity of LO is observed, particularly with longer reaction times and temperatures ≥ 100 °C. This indicates increased intensity of the main reaction and reduced participation of competing side reactions. At the highest tested content (10 wt%, Figure 9f), maximum LO selectivity values are observed, especially under high temperatures and at extended reaction times. This indicates the surface saturation effect on the catalyst and optimal conditions for the activity of catalytic sites.

The third group of plots (Figure 9g–i) shows the effect of temperature. At the low temperature of 50 °C (Figure 7g), the reaction proceeds very slowly with the low selectivity, likely due to insufficient energy to activate the substrate. At 100 °C (Figure 9h), a marked improvement in selectivity is observed, suggesting conditions approaching the kinetic optimum. At 150 °C (Figure 9i), maximum selectivity is achieved—the reaction proceeds almost entirely along the desired pathway, and these conditions promote full utilization of catalyst activity without the thermal degradation.

The analysis results clearly indicate that reaction temperature is the most influential factor affecting the selectivity toward linalool (LO), surpassing the effects of both catalyst content and reaction time. Higher temperatures not only accelerate the isomerization process but also promote pathways leading to increased LO formation. The presence of a greater amount of catalysts further enhances this effect by increasing the number of available active sites. Reaction time, while still important, primarily supports the process by allowing sufficient duration for the isomerization to proceed efficiently. By optimizing these three parameters, high geraniol conversion and favorable selectivity toward LO can be achieved, making the system both efficient and reliable. The developed statistical model can therefore serve as a valuable tool for guiding further process design and optimization.

#### 2.3.3. Influence of Process Parameters on DCM Selectivity

The presented study focused on evaluating the catalytic activity of vermiculite in the decomposition process of DCM and analyzing the impact of three key parameters—temperature, catalyst content, and reaction time—on the selectivity of this compound. To accurately characterize the studied relationships, a second-order multiple regression model and analysis of variance (ANOVA) were applied, with a statistical significance level of 95% (α = 0.05).

The developed statistical model showed excellent fit to the experimental data. This is confirmed by the high determination coefficient values: R^2^ reached 99.44%, the adjusted R^2^ was 99.15%, and the predicted R^2^ was 98.59%. Such a high level of agreement indicates that the independent variables (parameters)—temperature, catalyst content, and time—explain the variability of the process selectivity to a very large extent (Table 8).

The ANOVA analysis allowed identifying which of the process factors have the greatest impact on the efficiency of DCM conversion. Temperature plays a decidedly dominant role, as confirmed by very high F-statistic values (2767.08) and a significance level of < 0.0001. Catalyst content also has a significant, though somewhat smaller, effect (F = 191.81), while reaction time was found to be significant as well, but its contribution to shaping selectivity was less pronounced (F = 65.68). The quadratic terms (relating to nonlinear relationships) did not reach statistical significance—this indicates that the studied relationships are mostly linear within the adopted range of variable values. The only significant interaction term was the temperature–content pair (*p* = 0.040), suggesting that their simultaneous change may have a synergistic effect on selectivity (Table 9).

The obtained regression model equation in real units takes the following form:

(3) $$S_{DCM}=-0.068+0.01902\;T+0.0201\;C+0.01177\;\tau +0.000024\;T^{2}+0.00278\;C^{2}\;-0.000055\;{\;\tau }^{2}+0.000306\;T\cdot C+0.000084\;T\cdot \tau \;-0.000288\;C\cdot \tau \;$$

where

*S_DCM_* is DCM selectivity [mol %],

*T* is temperature [°C],

*C* is catalyst content [wt%],

*τ* is reaction time [h].

Analysis of the coefficients indicates that both temperature and catalyst content strongly promote the increase in reaction selectivity. The effect of reaction time is also noticeable; however, its significance decreases with longer reaction duration. Quadratic and interaction terms have a limited impact, suggesting a relatively simple course of catalytic phenomena within the considered conditions.

**Figure 10:**
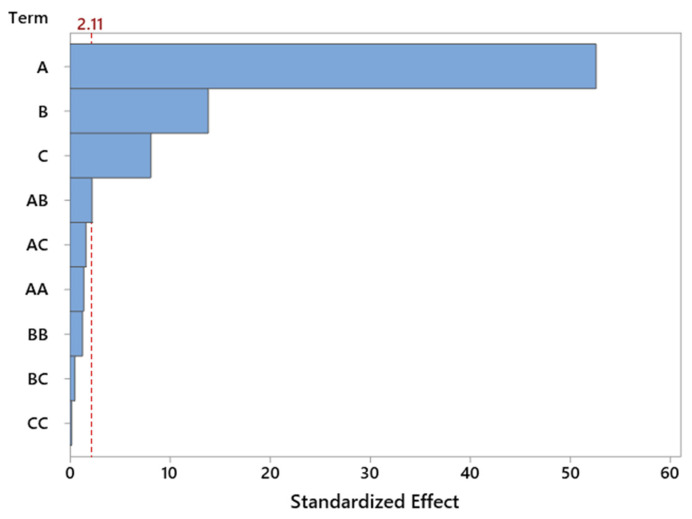
Pareto chart of the standardized effects of DCM selectivity plot for α = 0.05 (A—Reaction temperature, B—Catalyst content (wt%), C—Reaction time (hours), AB—Interaction between reaction temperature and catalyst content, AC—Interaction between reaction temperature and reaction time, BC—Interaction between catalyst content and reaction time, AA—Quadratic effect of reaction temperature (nonlinear effect of temperature), BB—Quadratic effect of catalyst content (nonlinear effect of catalyst content), CC—Quadratic effect of reaction time (nonlinear effect of reaction time)).

The use of a Pareto chart enabled a quick comparison of the strength of influence of individual factors. The chart clearly indicates that temperature is the dominant parameter—significantly exceeding the others in terms of its effect on selectivity. Catalyst content was also important to a lesser, but still noticeable, extent (Figure 10).

**Figure 11:**
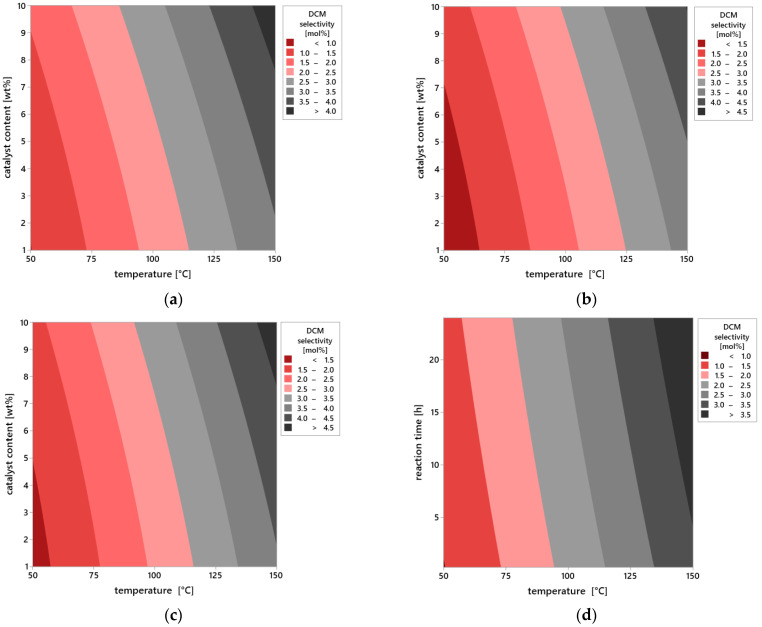

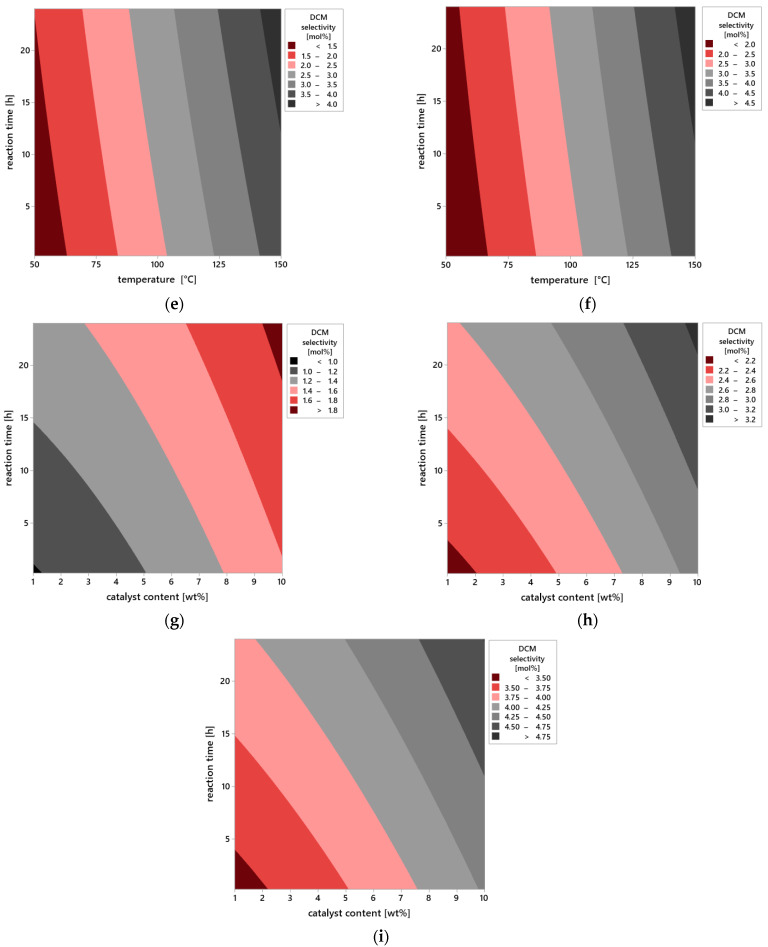
Contour plot of DCM selectivity at: time (**a**) 0.25 h, (**b**) time 12 h, (**c**) 24 h, catalyst content (**d**) 1 wt%, (**e**) 5 wt%, (**f**) 10 wt%, temperature (**g**) 50 °C, (**h**) 100 °C, (**i**) 150 °C.

Additional information on the influence of process parameters on the selectivity of DCM is provided by the analysis of contour plots (Figure 11a–i), which allow assessment of the selectivity distribution as the function of two operational variables while maintaining the third parameter at the constant level. This graphical representation of data enables a more precise understanding of the interdependencies between temperature, catalyst content, and reaction time, as well as their impact on selectivity efficiency.

In the first group of plots (Figure 11a–c), the effect of varying reaction time was analyzed while keeping the other parameters constant. For the shortest reaction time of 0.25 h (Figure 11a), selectivity remains at a very low level across the entire range of analyzed temperatures (50–150 °C) and catalyst content (1–10 wt%). The absence of distinct areas of increased selectivity indicates insufficient contact time between reagents and the active surface of vermiculite. Under this time frame, the catalytic system is unable to effectively carry out the DCM selectivity, regardless of intensification of the other variables.

As the reaction time is extended to 12 h (Figure 11b), a clear increase in selectivity is observed, particularly under conditions of elevated temperature and higher catalyst content. The plot reveals extensive iso-selectivity regions above 10 mol%, whose positions shift toward higher values of both variables. This observed effect suggests the existence of synergy between temperature and catalyst amount, which enhances the selective nature of the DCM selectivity.

The highest selectivity values are achieved after 24 h of reaction (Figure 11c). In this case, the plot indicates a distinct optimum area located around 150 °C and a catalyst content close to 10%. Selectivity in this region exceeds 20 mol%, meaning these conditions ensure near-maximum utilization of the catalytic potential of vermiculite. At the same time, noticeable flattening of contours at lower temperature and catalyst content values confirms the limited effectiveness of the reaction under less intense operating conditions, even with prolonged reaction time.

The second series of plots (Figure 11d–f) presents the influence of catalyst content at constant reaction time and variable temperature. For the lowest content—1 wt% (Figure 11d)—selectivity remains limited throughout the temperature range. The dominant area on the plot is below 10 mol%, indicating that a small amount of catalyst is insufficient to effectively carry out the reaction regardless of temperature increase. The situation improves at 5 wt% content of the catalyst (Figure 11e), where the first areas of moderate selectivity appear, reaching values between 10 and 15 mol%, especially at temperatures above 100 °C. At the maximum catalyst content—10 wt% (Figure 11f)—there is a significant expansion of high selectivity areas, concentrated at the highest temperatures. This figure clearly indicates that the availability of active sites on the catalyst surface is a limiting factor in the process, and increasing them directly translates into selectivity efficiency.

The final set of plots (Figure 11g–i) illustrates the effect of temperature at constant catalyst content and reaction time. At a low temperature of 50 °C (Figure 11g), regardless of other variables, the reaction proceeds with very low selectivity. Increasing the temperature to 100 °C (Figure 11h) results in a noticeable improvement—the increase in areas of moderate selectivity is visible, especially at higher catalyst contents and longer reaction times. Only at 150 °C (Figure 11i) are selectivity values exceeding 20 mol% obtained, indicating that this temperature level represents the optimum point in terms of the catalytic system’s activity.

In summary, the contour plots unequivocally confirm the results obtained from the statistical analysis. All the examined parameters—reaction time, catalyst content, and temperature—affect the selectivity of the DCM selectivity; however, temperature remains the decisive factor determining the overall process efficiency. Its influence is evident both under short reaction times and at lower catalyst content. The graphical results fully correspond with the F-statistic values and significance indicators, and their interpretation allows for precise identification of optimal conditions to maximize selectivity using vermiculite as the heterogeneous catalyst.

#### 2.3.4. Influence of Process Parameters on TH Selectivity

The conducted study focused on evaluating the influence of process parameters—temperature, catalyst content, and reaction time—on the selectivity of TH, using vermiculite as the heterogeneous catalyst. A second-order regression model was applied to describe the relationship between the independent variables and selectivity of TH, and its validity was assessed using analysis of variance (ANOVA) and confirmed by the graphical analysis of response surfaces. The statistical model showed very high agreement with the experimental data, as confirmed by the fit indicators: the determination coefficient R^2^ reached 98.95%, the adjusted R^2^ was 98.39%, while the predicted R^2^ was 97.23%. Such a high quality of fit indicates a strong dependence between the operational variables and the selectivity of TH (Table 10).

The presented Table 11 shows the results of the analysis of variance (ANOVA) for the model describing the effect of three independent variables—temperature, catalyst content, and reaction time—on the selectivity of TH. The overall model is statistically significant (*p* < 0.001), indicating the good fit to the data (F = 177.74). Among the model terms, the linear effects of all three factors are statistically significant (*p* = 0.000), with temperature having the largest contribution to the explained variance (Adjusted Sum of Squares = 1044.46). The quadratic effects of the variables are not significant (*p* > 0.3), suggesting a predominantly linear relationship within the tested range. Two-way interactions are significant overall (*p* = 0.042), but only the interaction between temperature and catalyst content is statistically meaningful (*p* = 0.019), indicating that the effect of one factor on TH selectivity depends on the level of the other. The other interactions (temperature–time and catalyst content–time) are not significant (*p* > 0.05). The error term has a small sum of squares (12.58) and low mean square (0.74), reflecting low unexplained variability and precise measurements. In summary, the model confirms that temperature and catalyst content are the main factors influencing TH selectivity, with their interaction playing a significant role, while the effect of time is less pronounced and nonlinear effects and other interactions are negligible in the studied range.

The obtained regression model equation in real units takes the following form:

(4) $$S_{TH}=-0.41+0.454\;T+0.04861\;C+0.01177\;\tau +0.000138\;T^{2}-0.0170\;C^{2}\;-0.00026{\;\tau }^{2}+0.00284\;T\cdot C+0.000772\;T\cdot \tau \;-0.00138\;C\cdot \tau \;$$

where

*S_TH_* is TH selectivity [mol %],

*T* is temperature [°C],

*C* is catalyst content [wt%],

*τ* is reaction time [h].

The model coefficients clearly indicate the dominant role of temperature as the factor determining the course of the reaction. Catalyst content also shows the significant, though somewhat smaller, influence, while reaction time has a moderate but still important effect. Quadratic and interaction terms have lower absolute values; however, the presence of a significant interaction between temperature and catalyst content suggests the possibility of a synergistic effect between these parameters.

Analysis of variance (ANOVA) confirmed the significance of all three main linear factors. Temperature proved to be the parameter with by far the greatest impact on the selectivity of the TH product, as reflected by an F-statistic value of 1411.02 and a significance level of *p* < 0.0001. Catalyst content also had a significant effect on the selectivity TH (F = 140.68; *p* < 0.0001), as did reaction time, although its influence was relatively smaller (F = 42.97; *p* < 0.0001). The quadratic terms, including T^2^, C^2^, and τ^2^, did not show statistical significance (*p* > 0.3), meaning that within the analyzed variable range, nonlinear effects on selectivity are negligible. Among the two-factor interactions, only temperature × catalyst content (T·C) reached significance (F = 6.67; *p* = 0.019), indicating the existence of an enhancing effect when these two parameters are increased simultaneously.

**Figure 12:**
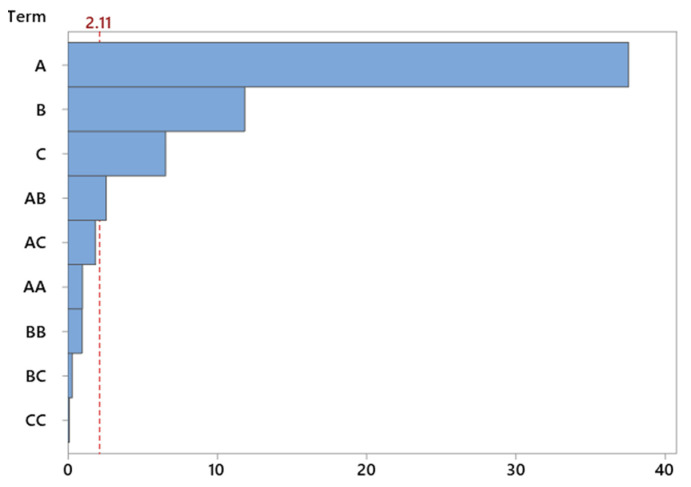
Pareto chart of the standardized effects of TH selectivity plot for α = 0.05 (A—Reaction temperature, B—Catalyst content (wt%), C—Reaction time (hours), AB—Interaction between reaction temperature and catalyst content, AC—Interaction between reaction temperature and reaction time, BC—Interaction between catalyst content and reaction time, AA—Quadratic effect of reaction temperature (nonlinear effect of temperature), BB—Quadratic effect of catalyst content (nonlinear effect of catalyst content), CC—Quadratic effect of reaction time (nonlinear effect of reaction time)).

The ANOVA results were complemented by a Pareto chart (Figure 12), which illustrates the relative strength of the influence of individual factors on TH selectivity. The chart clearly confirms the dominant role of temperature—it is the parameter that distinctly exceeds the others both in terms of direct impact and normalized effect. Catalyst content ranks second, while reaction time and interaction components are of secondary importance.

**Figure 13:**
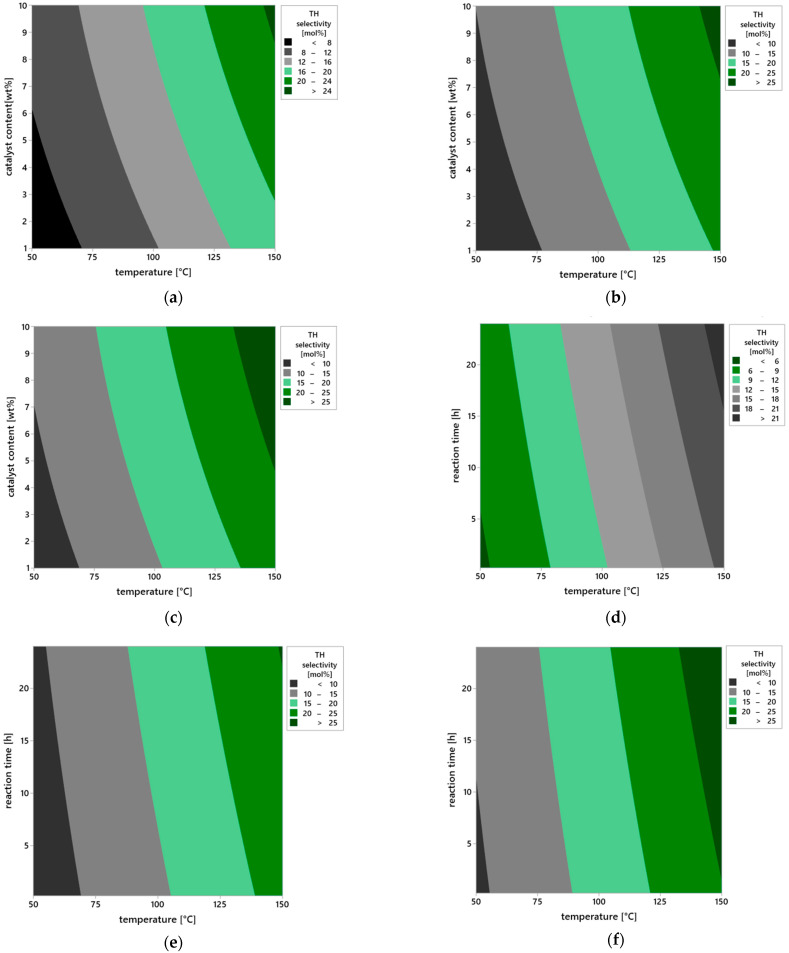

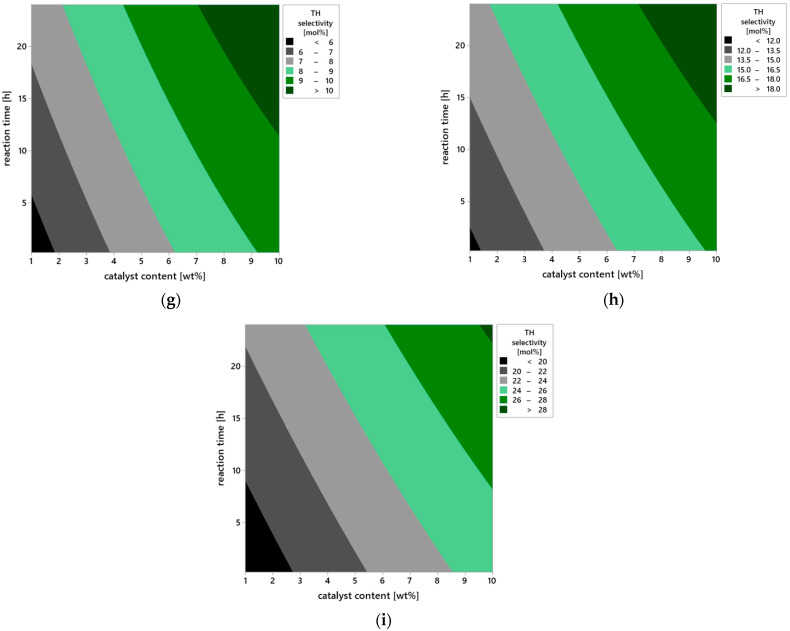
Contour plot of TH selectivity at: time (**a**) 0.25 h, (**b**) time 12 h, (**c**) 24 h, catalyst content (**d**) 1 wt%, (**e**) 5 wt%, (**f**) 10 wt%, temperature (**g**) 50 °C, (**h**) 100 °C, (**i**) 150 °C.

Further insights into the influence of operational parameters on the selectivity of the transformation leading to the TH product were provided by the analysis of contour plots (Figure 13a–i), which illustrate changes in TH selectivity values as a function of pairs of variables: temperature, catalyst content, and reaction time. This approach enables capturing not only the direct effects of individual factors but also their mutual interactions, which is particularly important in heterogeneous catalysis systems, where the balance between surface kinetics and reaction thermodynamics determines the direction of the transformation.

In the case of a short reaction time of 0.25 h (Figure 13a), the selectivity of TH product remains low across the entire range of studied temperatures (50–150 °C) and catalyst contents (1–10 wt%). This condition indicates that the catalytic mechanism does not fully develop within this time—substrates molecules have limited contact with the catalyst surface, and the formation of transition states or active chemisorbed complexes proceeds inefficiently. Under such short retention time conditions, the substrate does not reach equilibrium with the solid phase, resulting in a dominance of physical adsorption mechanisms, without deep involvement of active vermiculite surface sites in the chemical transformation step. Extending the reaction time to 12 h (Figure 13b) leads to a clear increase in TH selectivity, especially at elevated temperature and higher catalyst loading. This suggests that the TH formation process follows a multi-step mechanism requiring stable intermediates that gradually convert into the final product. In such a system, reaction time affects not only substrate conversion but also the equilibrium among intermediate products—prolonged exposure to the active surface favors the selective transformation of molecules into the thermodynamically more stable TH product.

After 24 h (Figure 13c), the system reaches the highest TH selectivity values, locally exceeding 25 mol%. The optimum is observed at 150 °C and 10 wt% catalyst content, confirming the synergistic interaction of these parameters. The presence of stable surface complexes on vermiculite, likely associated with hydroxyl groups or Brønsted/Lewis acid sites, allows prolonged catalytic activity without loss of selectivity, indicating the catalyst’s high structural and chemical stability.

The analysis of plots in Figure 13d–f, showing the effect of catalyst content under varying temperature and time conditions, provides information about the availability of active surface sites. For a low content of the catalyst—1 wt% (Figure 13d), regardless of temperature and reaction time, selectivity remains below 10 mol%. This is due to the limited number of catalytic active sites capable of adsorbing and activating substrate molecules—the deficit of active centers results in partial reaction progression and low TH selectivity. At catalyst content amounted to 5 wt% (Figure 13e), TH selectivity noticeably improves, particularly under longer reaction times and temperatures above 100 °C. It can be assumed that at this content, a sufficient number of active sites is formed, enabling both substrate adsorption and stabilization of transition states. Maximum TH selectivity values are observed at the catalyst content amounted to 10 wt% (Figure 13f), where a fully developed catalytic structure forms on the solid surface, and the reaction proceeds with high efficiency, reaching values exceeding 30 mol% at 150 °C and 24 h reaction time.

The plots in Figure 13g–i illustrate the direct influence of reaction temperature on TH selectivity in the context of varying catalyst content and reaction time. At the lowest tested temperature of 50 °C (Figure 13g), selectivity is low regardless of other parameters, clearly indicating insufficient substrate activation energy under weak heating conditions. Under such circumstances, both product desorption and transformation of chemisorbed intermediate forms are limited. Increasing the temperature to 100 °C (Figure 13h) results in a marked increase in TH selectivity—at higher catalyst contents and longer reaction times, values above 20 mol% are obtained. This suggests that within this temperature range, the reaction proceeds according to the thermal activation mechanism, and the presence of active vermiculite sites allows controlled steering of the reaction pathway toward the desired product. At 150 °C (Figure 13i), the catalyst achieves full activity, and the reaction system operates at maximum TH selectivity. The observed plateau of values above 30 mol% suggests the establishment of an equilibrium state in the isomerization process, leading to the formation of TH as a thermodynamically favored product under these conditions.

In summary, the analysis of contour plots from a chemical perspective indicates that the selectivity toward TH depends on the availability of active surface sites (controlled by catalyst content), the thermal activation of substrates (temperature), and the time required for multi-step rearrangements to proceed. Particularly important are potential electrophilic interactions and stepwise isomerization pathways, in which the active surface of vermiculite not only adsorbs but also stabilizes reactive intermediates. This behavior is characteristic of materials with acidic sites and layered structures that facilitate substrate migration and concentration. The confirmed high selectivity toward TH and the stability of the catalytic structure under the tested reaction conditions clearly indicate that vermiculite is a promising material for further applications in selective heterogeneous catalysis, particularly in terpene isomerization processes and the synthesis of oxygenated compounds such as TH.

## 3. Discussion

The conducted studies analyzed the influence of three key reaction parameters—temperature, catalyst content, and process duration—on the conversion of geraniol (GA) and the selectivity of the resulting reaction products: linalool (LO), thunbergol (TH), and 6,11-dimethyl-2,6,10-dodecatrien-1-ol (DCM). These products differ both in their structural complexity and in the energy requirements for their formation, which was reflected in the experimental results and confirmed the presence of a multi-step reaction mechanism involving reactive carbocation intermediates [43,44].

The isomerization of geraniol in the presence of natural vermiculite is an example of a reaction occurring on the surface of a layered acidic catalyst, where both the substrate structure and the properties of the catalytic center play key roles [5,37]. Geraniol, an unsaturated terpenoid alcohol (C_10_H_18_O), contains a conjugated bonding system and a hydroxyl group that easily undergoes protonation [7,19]. Mechanistically, the reaction is initiated by protonation of the –OH group by Brønsted acid centers present on the vermiculite surface, leading to the formation of an allylic carbocation with an extensive resonance system [42]. This carbocation can undergo rotations, rearrangements, electrocyclic reactions (leading to TH) [36], condensations (leading to DCM), or simple isomerizations leading to LO [35].

The analysis of temperature influence showed that increasing temperature from 50 °C to 80 °C results in a significant increase in GA conversion (from 84.6% to 99.6%) and LO selectivity, reaching a maximum (15.81%) at 70 °C [40]. The preferential formation of linalool within this temperature range may be related to the relatively low activation energy for hydroxyl isomerization and the stability of LO under moderate conditions [35]. The low selectivity of TH and DCM in this range suggests that their formation requires conditions more favorable for multi-step rearrangements or cyclizations, which are kinetically and thermodynamically less favored at lower temperatures [36]. Optimization studies conducted over a broader temperature range (up to 140 °C) showed that temperature becomes the dominant factor promoting the formation of more complex products—primarily thunbergol—indicating a shift in the mechanism towards cyclization reactions [40].

Catalyst content also had a clear impact on product profiles and system activity. Even at 1 wt% vermiculite, significant GA conversion (89.12%) and high TH selectivity (26.48%) were observed, indicating a high density of acid active sites on the mineral surface [37,42]. Increasing the catalyst content to 10% resulted in TH selectivity of 65.18%, LO 24.77%, and DCM 2.06% [43]. Increasing the catalyst content raises the number of Brønsted and Lewis acid centers, which enhances the intensification of cyclization, dehydration, and condensation pathways [5]. A high surface charge density facilitates substrate protonation and stabilization of intermediate forms—especially cyclic carbocations leading to TH and DCM, whose formation is associated with rearrangements and coupling of unsaturated systems [36].

The reaction time was equally important, regulating the transformation dynamics. Within the first 15 min, GA conversion reached 99.52%, with simultaneously low LO selectivity (5.7%) and moderate TH (14.62%) [40]. With prolonged reaction time, the transformation evolved—selectivity of both LO and TH increased, reaching maxima (24.77% and 65.96%) after about 3 h [43]. Further extending the process led to a decrease in the identified products, which can be associated with their secondary transformations, including polymerization or degradation [44]. These phenomena indicate the necessity of optimal balancing of conditions: too short a time prevents full conversion of the substrate to desired isomers, while too long can cause irreversible losses due to secondary reactions [43].

The structure of vermiculite—consisting of tetrahedral (SiO_4_) and octahedral (Mg^2+^, Fe^3+^, Al^3+^) layers located in interlayer spaces—provides a large active surface, swelling capability, presence of Lewis and Brønsted acid centers, and sorptive properties [37,38]. Adsorption of organic molecules in the layered channels promotes the formation of local reaction environments (so-called microreactors), which can increase selectivity by limiting the rotational freedom of intermediates and stabilizing transition states [37].

In the conducted studies, the potential of natural vermiculite as a heterogeneous catalyst for the isomerization of geraniol (GA) was examined, and reaction conditions were optimized to maximize substrate conversion and selectivity towards the three main products: linalool (LO), thunbergol (TH), and 6,11-dimethyl-2,6,10-dodecatrien-1-ol (DCM) [40,42]. A statistical experimental design approach using the response surface methodology (RSM) was applied, allowing not only precise description of the influence of three operational variables (temperature, catalyst content, and reaction time) but also identification of their interactions and nonlinear effects [43].

Statistical models describing selectivity of each product showed very high fit quality. For example, for LO selectivity, the determination coefficient was R^2^ = 99.56%, and adjusted R^2^ = 99.33%, indicating an almost perfect match between the model and experimental values. Similar values were obtained for TH and DCM selectivity. High predictive power (R^2^ pred > 98%) confirmed the model’s usefulness for predicting results beyond the measurement range, indicating high process stability and feasibility for further technological scaling [43].

Analysis of variance (ANOVA) revealed that all three linear factors—temperature, catalyst content, and reaction time—have a significant impact on the process course (*p* < 0.0001) [43]. Temperature was identified as the dominant factor: the F-statistic value for this parameter exceeded 3400, indicating an extremely strong dependence between temperature and product selectivity. Catalyst content also had a significant, though somewhat weaker, effect (F ≈ 220), while reaction time showed statistically significant but less important influence (F ≈ 126), suggesting it is primarily relevant for optimizing selectivity rather than conversion itself [43].

Quadratic and interaction factors were also included in the models, allowing identification of nonlinearities and synergistic effects. The most significant among quadratic factors was temperature (F = 52.09), confirming that its effect on selectivity is not linear—temperature increases beyond a certain threshold shift the reaction equilibrium towards cyclization and condensation [40]. In contrast, the quadratic term for reaction time was statistically insignificant (*p* = 0.987), indicating that beyond a certain value no further selectivity changes occur—the reaction reaches a quasi-stationary state [40]. Most two-factor interactions were not statistically significant, except for the interaction between temperature and reaction time (*p* = 0.004), which might indicate a dependence of product type on the duration of substrate exposure to high-temperature conditions [43].

Graphical analysis of the results in the form of contour plots additionally highlighted the nonlinear nature of selectivity changes as a function of the analyzed variables. For example, LO selectivity sharply increased at medium temperatures and low catalyst concentrations, while TH selectivity dominated at the highest temperatures and high catalyst content [40]. For DCM, relative stability was observed in a narrow parameter range, possibly indicating its formation as a by-product of parallel or secondary reactions [44].

In summary, applying the RSM method enabled: identification of parameter ranges leading to maximized yields of desired products, reduction in the number of necessary experiments, and creation of a predictive tool useful for further process scale-up [43]. This allowed for clear determination that temperatures of 130–140 °C, vermiculite content of 10%, and reaction time of 3 h represent optimal conditions at which both maximum GA conversion and highest selectivity toward thunbergol were achieved [43].

## 4. Materials and Methods

### 4.1. Raw Materials

The syntheses were carried out using vermiculite (100% pure, from Bauwer, Boguchwała, Poland) as the catalyst. The organic raw material employed in this study was GA (99% pure, sourced from Acros Organics, Geel, Belgium). For quantitative analysis, performed using gas chromatography (GC), the following standards were applied: citronellol (95% pure, from Sigma-Aldrich, Steinheim, Germany), citral (95% pure, from Sigma-Aldrich, Steinheim, Germany), ocymene (90% pure, from Sigma-Aldrich, St. Louis, MO, USA), β-pinene (95% pure, from Fluka, Buchs, Switzerland), linalool (97% pure, from Acros Organics, Geel, Belgium), farnesol (96% pure, from Acros Organics, Geel, Belgium), nerol (97% pure, from Acros Organics, Geel, Belgium), myrcene (technical grade, from Sigma-Aldrich, Steinheim, Germany), and geranylgeraniol (85% pure, from Sigma-Aldrich, St. Louis, MO, USA).

### 4.2. Characteristics of Vermiculite with Instrumental Methods

Comprehensive characterization of the vermiculite sample was performed using X-ray diffraction (XRD), Fourier-transform infrared spectroscopy (FTIR), and scanning electron microscopy (SEM) with energy-dispersive X-ray spectroscopy (EDS).

Structural analysis with XRD method was performed using an EMPYREAN II X-ray diffractometer (PANalytical, Almelo, The Netherlands). A copper lamp with a graphite monochromator was used as the radiation source, ensuring high purity of the radiation beam. Measurements were conducted with a counting time of 150.45 s per measurement point and a goniometer step size of 0.022°. The measurement angular range covered 10° to 50° 2θ. Phase identification was carried out based on data from the current PDF–4+ 2024 database.

Studies with FTIR method were performed using a Nicolet iS5 spectrophotometer (ThermoFisher, Waltham, MA, USA) over the wavenumber range of 1500 to 300 cm^−1^, enabling detailed analysis of characteristic functional groups present in the sample of vermiculite.

SEM method was used to examine the morphology of the vermiculite sample with a JEOL JSM-6490 LV scanning electron microscope (JEOL Ltd., Tokyo, Japan) equipped with an energy-dispersive X-ray spectrometer (EDS) for micro-area chemical analysis. The vermiculite samples were first cooled in liquid nitrogen, then fractured with a hammer to expose fracture surfaces for observation. The sample surfaces were coated with a thin layer of gold (approximately 10 nm) by sputtering using a JEOL JFC-1300 device (JEOL Ltd., Tokyo, Japan), which prevented the accumulation of electric charges during measurement and enabled the acquisition of clear micrographs showing morphology and surface structure.

### 4.3. Method of Carrying out Isomerization of Geraniol on Vermiculite and Methods of Analysis of the Post-Reaction Mixtures

The syntheses were conducted in a glass reactor with a volume of 25 cm^3^, equipped with a reflux condenser and a magnetic stirrer with heating capability. The studied parameters covered the following ranges: temperature from 80 to 150 °C, catalyst content between 5 and 15 wt %, and reaction time from 15 min to 24 h. To perform both qualitative and quantitative analyses, each post-reaction mixture was first centrifuged, and the resulting sample was dissolved in acetone in the 1:3 ratio.

Qualitative analysis was conducted using GC-MS method on a Schimadzu instrument with a Voyager detector and a DB-5 column (containing phenylmethylsiloxanes, 30 m × 0.25 mm × 0.5 mm). The analyses conditions were as follows: helium flow rate of 1 mL/min, sample chamber temperature set at 200 °C, detector temperature at 250 °C, and an oven temperature that remained isothermal for 2.5 min at 50 °C, followed by a ramp at a rate of 10 °C/min up to 300 °C.

For quantitative analysis, a Schimadzu chromatograph with an FID detector and a TR-FAME column (cyanopropylphenyl packed, 30 m × 0.25 mm × 0.25 mm) was used. The analyses parameters included a helium flow rate of 0.7 mL/min, sample chamber temperature at 200 °C, detector temperature at 250 °C, and oven temperature set to 60 °C for 7 min, then heated at a rate of 15 °C/min to 240 °C. The FID temperature was maintained at 250 °C.

The area normalization method was used for quantitative analysis, with an estimated GC method error of approximately 2%. Prior to qualitative and quantitative analyses, samples of the post-reaction mixtures were diluted with acetone at a ratio of 1:5. After chromatographic analysis, the mass balance was calculated for each synthesis. This was achieved by first determining the peak areas of the individual compounds. The peak area of each compound was then divided by the sum of all peak areas, and the concentration of each compound, expressed as weight percent, was calculated. Based on the known mass of the post-reaction mixture, the mass of the individual compounds was calculated in grams and subsequently converted into moles. Next, we calculated the number of moles of geraniol that reacted to form compounds other than those we were able to identify in the post-reaction mixture (a value required to determine the total selectivity of the transformation into compounds that could not be individually quantified). For this purpose, the sum of the moles of the identifiable compounds was subtracted from the number of moles of geraniol that had reacted. Finally, we calculated the selectivities of the respective products as well as the geraniol conversion.

The following main process functions were calculated to characterize each synthesis:

Conversion of GA:

(5) $$Conversion\;of\;GA\left(\%\right)=\frac{Number\;of\;moles\;of\;reacted\;geraniol}{Initial\;number\;of\;moles\;of\;geraniol}\;\times 100$$

Selectivity of appropriate product:

(6) $$Selectivity\;of\;product\left(\%\right)=\frac{Number\;of\;moles\;of\;product}{Number\;of\;moles\;of\;reacted\;geraniol}\;\times 100$$

### 4.4. Selection of Process Parameters

The process parameters and their variation ranges were selected based on our previous studies as well as the available literature data related to the catalytic transformation of geraniol and similar terpene compounds. The selected factors—temperature, catalyst content, and reaction time—are known to significantly influence both the conversion of the substrate and the selectivity towards desired products.

The temperature range (50–150 °C) was chosen to cover both mild and moderate thermal conditions, enabling the evaluation of reaction progress at lower energy input, as well as assessing possible side reactions or thermal degradation at elevated temperatures.

The catalyst content (1–10 wt%) was varied to determine the influence of the catalyst load on the reaction efficiency. The lower limit reflects minimal catalytic activity, while the upper value allows us to study potential saturation effects or diffusion limitations.

The reaction time (0.25–24 h) was selected to monitor the kinetics of product formation, from the early stages of the reaction to prolonged conditions that may favor secondary transformations or product degradation.

The three-level factorial design enabled the systematic evaluation of how each factor affects both the conversion of geraniol and the selectivity of the target products. The control levels used in this study are summarized in Table 12.

The Design of Experiments (DOE) was employed to reduce the number of tests and minimize inspection time. The experiments were conducted according to the full factorial design. To analyze the data, the Response Surface Method (RSM) was applied using the central composite model, consisting of 27 experiments (Table 13). RSM integrates statistical and mathematical approaches to modeling, considering the relationship between the individual variables of the process and the observed responses [44]. The second-degree multinomial equation used to determine the regression model value is presented below:

(7) $$y=\beta_{0}+\sum_{i=1}^{k}\beta_{i}x_{i}+\sum_{i=1}^{k}\beta_{ii}x_{i}^{2}\pm \varepsilon$$

where

- y is the dependent variable (response),

- x_i_ indicates values of the i-th parameter,

- β_0_, β_i_, β_ii_ are the coefficients of regressions,

- ε is the error acquiring.

**Table 13:** Parameters of individual syntheses (experimental plan) and their corresponding response function values.

Synthesis Number	Temperature	Vermiculite Content	Time	GA Conversion	LO Selectivity	DCM Selectivity	TH Selectivity	Carbon Balance
*-*	[°C]	[wt%]	[h]	[% mol]	[% mol]	[% mol]	[% mol]	[%]
1	50	1	0.25	84.0	5.0	1.0	6.0	30.00
2	50	1	12	85.2	6.0	1.2	7.2	32.28
3	50	1	24	85.4	7.0	1.4	8.0	34.00
4	50	5	0.25	85.0	7.5	1.3	7.5	33.00
5	50	5	12	85.2	8.5	1.4	8.5	36.46
6	50	5	24	85.4	9.0	1.5	9.0	38.50
7	50	10	0.25	85.6	8.5	1.5	9.0	37.50
8	50	10	12	85.8	10.0	1.6	9.5	40.78
9	50	10	24	86.0	12.0	1.8	10.0	43.80
10	100	1	0.25	85.4	15.0	2.0	10.0	52.00
11	100	1	12	87.2	18.0	2.2	12.0	57.80
12	100	1	24	88.0	20.0	2.5	14.0	63.50
13	100	5	0.25	88.0	20.5	2.4	14.5	64.86
14	100	5	12	89.0	22.0	2.6	15.5	67.64
15	100	5	24	90.0	25.0	2.8	17.0	72.92
16	100	10	0.25	90.0	22.5	3.0	17.5	71.70
17	100	10	12	91.0	25.0	3.2	19.0	76.48
18	100	10	24	92.0	28.0	3.4	20.5	81.76
19	150	1	0.25	90.6	25.0	3.5	19.5	78.30
20	150	1	12	92.2	28.0	3.8	21.0	83.12
21	150	1	24	93.2	30.0	4.0	22.5	87.40
22	150	5	0.25	91.6	28.5	3.7	22.0	86.08
23	150	5	12	92.6	31.0	4.0	23.5	91.00
24	150	5	24	93.6	34.0	4.2	25.0	96.28
25	150	10	0.25	92.8	30.0	4.2	24.0	91.08
26	150	10	12	93.8	33.0	4.5	26.0	97.50
27	150	10	24	94.4	36.0	4.7	28.0	99.04

Temp.—temperature; GA—geraniol, LO- linalool, DCM—6,11-dimethyl-2,6,10-dodecatrien-1-ol, TH—thunbergol; Total number of carbon moles at the inlet: C_in_ = F_A0_ ⋅ n_c,a_, Total number of carbon moles at the outlet: C_out_ = (F_A_ ⋅ n_c,a_) + ∑C_i_, Carbon balance: % Balance = C_out_/C_in_ ⋅ 100%; F_A0_—initial molar flow of geraniol (at the inlet, before the reaction), n_C,i_—number of carbon atoms in one molecule of product i, F_A_—molar flow of geraniol remaining after the reaction (unreacted), C_in_—total carbon entering the system, C_out_—total carbon leaving the system [mol C], % Balance—percentage closure of the carbon balance.

The Minitab 21.4.3.0 Statistical Software was utilized for the computation of the model equations. The study results, detailing the influence of process control parameters (independent variables) on geraniol (GA) conversion and the selectivities of linalool (LO), 6,11-dimethyl-2,6,10-dodecatrien-1-ol (DCM), and thunbergol (TH) (dependent variables), are presented in Table 2. In Table 2, columns 2 to 4 list the values of the process control parameters (input variables) for the studied conditions, while columns 5 to 7 display the response function values (output variables).

The five response functions used in the optimization studies were selected based on preliminary screening experiments and literature data indicating their significance for product yield and quality.

Among the studied selectivities, linalool and thunbergol exhibited the highest values. Although the selectivity of DCM was relatively low, this compound was included in the optimization due to its importance as a valuable intermediate in the synthesis of complex terpenoids and its potential applications in fragrance and pharmaceutical industries. These applications and the relevance of DCM and thunbergol are discussed in detail in the Introduction section to justify their selection despite the lower selectivity of DCM.

Thus, the choice of these three compounds for optimization was driven both by their abundance in the reaction mixture and their practical significance.

## 5. Conclusions

The present study demonstrates that natural vermiculite is an effective and environmentally friendly heterogeneous catalyst for the isomerization of geraniol, achieving moderate conversion while maintaining high selectivity toward the main products, namely linalool, thunbergol, and 6,11-dimethyl-2,6,10-dodecatrien-1-ol. Structural characterization confirmed that vermiculite possesses a layered architecture, high specific surface area, and a combination of Brønsted and Lewis acid sites, which are responsible for its catalytic performance.

Optimization of key reaction parameters—temperature, catalyst content, and reaction time—showed that moderate reaction conditions (approximately 70–140 °C, 5–10 wt% catalyst, 120–180 min) provide an optimal balance between conversion and selectivity, while minimizing energy consumption compared to more demanding catalysts such as diatomite or modified halloysite. Temperature was identified as the most influential factor affecting both conversion and product distribution, followed by catalyst content and reaction time, highlighting the importance of careful control of reaction conditions.

Vermiculite demonstrated the ability to selectively favor the formation of desired geraniol isomers under mild conditions, in contrast to other catalysts which often require higher temperatures, longer reaction times, or specific modifications to achieve comparable selectivity. This indicates that vermiculite is not only effective but also suitable for sustainable chemical processes from both economic and ecological perspectives.

The study confirms that natural vermiculite can serve as a promising alternative to more expensive and energy-intensive catalysts, combining high selectivity, moderate conversion, low cost, and reduced environmental impact. Future work should focus on enhancing the catalytic activity of vermiculite, for example, through acid treatment, as well as evaluating its long-term stability and recyclability under repeated reaction cycles to fully assess its potential for industrial applications in green chemistry.

## Figures and Tables

**Table 1:** Effect of temperature on the course of geraniol transformation in the presence of vermiculite as the catalyst.

Synthesis Number	Temperature	Vermiculite Content	Time	LO Selectivity	DCM Selectivity	TH Selectivity	GA Conversion
	[°C]	[wt%]	[h]	[mol %]	[mol %]	[mol %]	[mol %]
1	50	5	3	3.95	1.66	5.60	84.60
2	60	5	3	6.35	2.96	10.77	84.39
3	70	5	3	15.80	2.82	37.70	91.98
4	80	5	3	15.73	2.01	36.03	99.60
5	90	5	3	Polymerization products			
6	100	5	3				
7	110	5	3				
8	120	5	3				
9	130	5	3				
10	140	5	3				
11	150	5	3				

Linalool (LO), 6,11-dimetylo-2,6,10-dodekatrien-1-ol (DCM), thunbergol (TH).

**Table 2:** Effect of the amount of catalyst (vermiculite) on the course of geraniol transformation process.

Synthesis	Temperature	Catalyst Content Vermiculite	Reaction Time	Selectivity of LO	Selectivity of DCM	Selectivity of TH	Conversion of GA
	[°C]	[wt%]	[h]	[mol %]	[mol %]	[mol %]	[mol %]
1	70	1.0	3	14.94	0.32	26.48	89.12
2	70	2.5	3	14.42	0.43	28.31	89.76
3	70	5.0	3	15.81	2.82	37.70	91.98
4	70	7.5	3	22.71	2.34	60.86	99.38
5	70	10.0	3	24.77	2.06	65.18	99.81

Linalool (LO), 6,11-dimetylo-2,6,10-dodekatrien-1-ol (DCM), thunbergol (TH).

**Table 3:** Effect of reaction time on the course of geraniol transformation in the presence of vermiculite as a catalyst.

Synthesis	Temperature	Catalyst Content	Reaction Time	Selectivity of LO	Selectivity of DCM	Selectivity of TH	Conversion of GA
	[°C]	[wt%]	[h]	[mol %]	[mol %]	[mol %]	[mol %]
1	70	10	0.25	5.7	0.04	14.62	99.52
2	70	10	0.50	13.28	0.48	53.29	99.54
3	70	10	1	19.73	0.73	54.1	99.56
4	70	10	2	20.74	1.66	57.89	99.81
5	70	10	3	24.77	2.06	65.96	99.81
6	70	10	4	Polymerization products			
7	70	10	5				
8	70	10	6				
9	70	10	24				

Linalool (LO), 6,11-dimetylo-2,6,10-dodekatrien-1-ol (DCM), thunbergol (TH).

**Table 4:** Model summary of geraniol conversion.

S	R-sq	R-sq (adj)	R-sq (pred)
0.735052	96.92%	95.28%	92.17%

**Table 5:** Analysis of variance of geraniol conversion.

Source	DF	Adj SS	Adj MS	F-Value	*p*-Value	VIF
Model	9	288.575	32.064	59.34	0.000	
Linear	3	286.312	95.437	176.64	0.000	-
Temperature [°C]	1	251.436	251.436	465.36	0.000	1.00
Catalyst content [wt%]	1	22.639	22.639	41.90	0.000	1.00
Time [h]	1	12.243	12.243	22.66	0.000	1.00
Square	3	0.215	0.072	0.13	0.939	-
Temperature [°C] × Temperature [°C]	1	0.027	0.027	0.05	0.827	1.00
Catalyst content [wt%] × Catalyst content [wt%]	1	0.004	0.004	0.01	0.936	1.00
Time [h] × Time [h]	1	0.185	0.185	0.34	0.566	1.00
2-Way Interaction	3	2.273	0.758	1.40	0.276	-
Temperature [°C] × Catalyst content [wt%]	1	0.410	0.410	0.76	0.396	1.00
Temperature [°C] × Time [h]	1	1.333	1.333	2.47	0.135	1.00
Catalyst content [wt%] × Time [h]	1	0.530	0.530	0.98	0.336	1.00
Error	17	9.185	0.540			
Total	26	297.760				

**Table 6:** Model summary of LO selectivity.

S	R-sq	R-sq (adj)	R-sq (pred)
0.810763	99.56%	99.33%	98.90%

**Table 7:** Analysis of variance of LO selectivity.

Source	DF	Adj SS	Adj MS	F-Value	*p*-Value	VIF
Model	9	2540.40	282.27	429.41	0.000	
Linear	3	2494.99	831.66	1265.20	0.000	-
Temperature [°C]	1	2267.51	2267.51	3449.54	0.000	1.00
Catalyst content [wt%]	1	144.59	144.59	219.97	0.000	1.00
Time [h]	1	82.93	82.93	126.16	0.000	1.00
Square	3	40.67	13.56	20.62	0.000	-
Temperature [°C] × Temperature [°C]	1	34.24	34.24	52.09	0.000	1.00
Catalyst content [wt%] × Catalyst content [wt%]	1	6.43	6.43	9.78	0.006	1.00
Time [h] × Time [h]	1	0.00	0.00	0.00	0.987	1.00
2-Way Interaction	3	9.29	3.10	4.71	0.014	-
Temperature [°C] × Catalyst content [wt%]	1	0.94	0.94	1.43	0.248	1.00
Temperature [°C] × Time [h]	1	7.52	7.52	11.44	0.004	1.00
Catalyst content [wt%] × Time [h]	1	0.83	0.83	1.26	0.277	1.00
Error	17	11.17	0.66			
Total	26	2551.57				

**Table 8:** Model summary of DCM selectivity.

Scheme	R-sq	R-sq (adj)	R-sq (pred)
0.107199	99.44%	99.15%	98.59%

**Table 9:** Analysis of variance of DCM selectivity.

Source	DF	Adj SS	Adj MS	F-Value	*p*-Value	VIF
Model	9	34.8654	3.8739	337,11	0.000	
Linear	3	34.7571	11.5857	1008.18	0.000	-
Temperature [°C]	1	31.7985	31.7985	2767.08	0.000	1.00
Catalyst content [wt%]	1	2.2043	2.2043	191.81	0.000	1.00
Time [h]	1	0.7548	0.7548	65.68	0.000	1.00
Square	3	0.0412	0.0137	1.20	0.341	-
Temperature [°C] × Temperature [°C]	1	0.0224	0.0224	1.95	0.181	1.00
Catalyst content [wt%] × Catalyst content [wt%]	1	0.0184	0.0184	1.60	0.222	1.00
Time [h] × Time [h]	1	0.0004	0.0004	0.03	0.862	1.00
2-Way Interaction	3	0.0898	0.0299	2.61	0.085	-
Temperature [°C] × Catalyst content [wt%]	1	0.0571	0.0571	4.97	0.040	1.00
Temperature [°C] × Time [h]	1	0.0299	0.0299	2.60	0.125	1.00
Catalyst content [wt%] × Time [h]	1	0.0028	0.0028	0.25	0.625	1.00
Error	17	0.1954	0.0115			
Total	26	35.0607				

**Table 10:** Model summary of TH selectivity.

S	R-sq	R-sq (adj)	R-sq (pred)
0.860356	98.95%	98.39%	97.23%

**Table 11:** Analysis of variance of TH selectivity.

Source	DF	Adj SS	Adj MS	F-Value	F-Value	*p*-Value
Model	9	1184.11	131.57	177.74	177.74	0.000
Linear	3	1180.38	393.46	531.55	531.55	0.000
Temperature [°C]	1	1044.46	1044.46	1411.02	1411.02	0.000
Catalyst content [wt%]	1	104.14	104.14	140.68	140.68	0.000
Time [h]	1	31.80	31.80	42.97	42.97	0.000
Square	3	1.41	0.47	0.63	0.63	0.603
Temperature [°C] × Temperature [°C]	1	0.71	0.71	0.96	0.96	0.341
Catalyst content [wt%] × Catalyst content [wt%]	1	0.69	0.69	0.93	0.93	0.348
Time [h] × Time [h]	1	0.01	0.01	0.01	0.01	0.919
2-Way Interaction	3	7.52	2.51	3.39	3.39	0.042
Temperature [°C] × Catalyst content [wt%]	1	4.93	4.93	6.67	6.67	0.019
Temperature [°C] × Time [h]	1	2.52	2.52	3.41	3.41	0.082
Catalyst content [wt%] × Time [h]	1	0.07	0.07	0.09	0.09	0.770
Error	17	12.58	0.74			
Total	26	1196.69				

**Table 12:** Control parameters and their values.

Studied Parameters	Unit	Values		
		1	2	3
Temperature	[°C]	50	100	150
Catalyst content	[wt%]	1	5	10
Time (reaction time)	[h]	0.25	12	24

## Data Availability

The data presented in this study are available upon request from the corresponding author.
